# Challenges for human oversight in detecting system malfunctions in AI-assisted medical diagnostics

**DOI:** 10.1038/s41746-026-03410-x

**Published:** 2026-11-13

**Authors:** Nadine Schlicker, Constanze Tepohl, Ali Kholmovaia, Noëlle Steffens, Niels van Berkel, Andreas Mayr, Ivica Grgic, Martin C. Hirsch, Markus Langer

**Affiliations:** 1 Philipps-Universität Marburg, Marburg, Germany; 2 Technical University of Munich, Munich, Germany; 3 Albert–Ludwigs–Universität Freiburg, Freiburg, Germany; 4 Aalborg University, Aalborg, Denmark

**Keywords:** Cardiology, Computational biology and bioinformatics, Health care, Mathematics and computing, Scientific community

## Abstract

Increasingly, physicians must oversee AI systems to detect system malfunctions. We studied 48 physicians in a randomized controlled study using an increasingly malfunctioning AI for cardiac auscultation. Diagnostic performance declined alongside AI reliability, even when physicians were cognitively forced to make an initial classification, and when human performance exceeded AI reliability. Physicians’ confidence declined faster than their trust in AI. Results call for specification of oversight goals and processes ensuring patient safety.

## Main Text

Consider an AI-system screening patients for cardiac conditions. For months, it has operated reliably, improving diagnostic accuracy and efficiency. Suddenly, a malfunction causes it to miss heart murmurs, producing erroneous outputs, ultimately posing risks to patient health.

Human oversight is often proposed as a safeguard against such risks^1,2^. Humans are expected to serve as a “safety net” by detecting inaccurate outputs and malfunctions, whether caused by technical errors, data drift, faulty inputs, or cyberattacks^3^. While research has examined how AI can augment human decision-making^4^, less attention has been directed to how reliably humans can prevent the consequences of system malfunctions.

Detecting system malfunctions is demanding, requiring sustained vigilance and cognitive engagement, especially when a system has previously performed flawlessly^5^. Automation bias may lead physicians to over-rely on AI recommendations, using AI “as a heuristic replacement for vigilant information-seeking and processing”^6^. Moreover, AI outputs can serve as cognitive anchors, inducing confirmation bias toward the initially presented outputs^2,7^, thereby reducing cognitive engagement and increasing the likelihood of adopting rather than questioning AI recommendations^8–11^. To counter such detrimental effects, cognitive forcing techniques have been proposed to maintain active engagement^2,5,12^, e.g., by requiring an independent assessment before revealing AI recommendations^12^. Such cognitive forcing requires physicians to actively process clinical data, develop hypotheses, and seek relevant evidence^2,9^. AI recommendations should then update rather than anchor physicians’ initial assessments^2^.

This study examined whether and when physicians can detect AI malfunctions, intervene appropriately, and whether cognitive forcing strategies can facilitate these tasks. We hypothesized that physicians who make an independent classification before, compared to after, receiving the AI recommendation would better detect and override inaccurate AI recommendations, and adjust their trust in the AI-system accordingly^5,12,13^. We additionally examined how this intervention affects physicians’ confidence in their own classifications^9,14^.

We conducted a randomized-controlled experiment using a mixed 2×4 design in the context of AI-assisted cardiac auscultation. Forty-eight physicians were randomly assigned to either an AI-First or AI-Second condition. Participants were informed about the AI’s training data and validation performance (96% accuracy with 97% sensitivity, 89% specificity). Each participant classified 40 digitally recorded heart sounds, presented across four blocks, as either normal or abnormal. To simulate system malfunction, system reliability declined stepwise across blocks (90%, 60%, 50%, 0%). In the final block, auditory and visual cues were deliberately mismatched to provide an additional signal of severe system malfunction^15^. The AI-Second condition implemented the cognitive forcing technique: Participants first provided an unassisted classification and confidence rating, which they could revise after receiving the AI recommendation. The AI-First condition received the AI recommendation before providing their classification and confidence rating. In both groups, participants rated their trust in the system after every fifth trial.

**Fig. 1:**
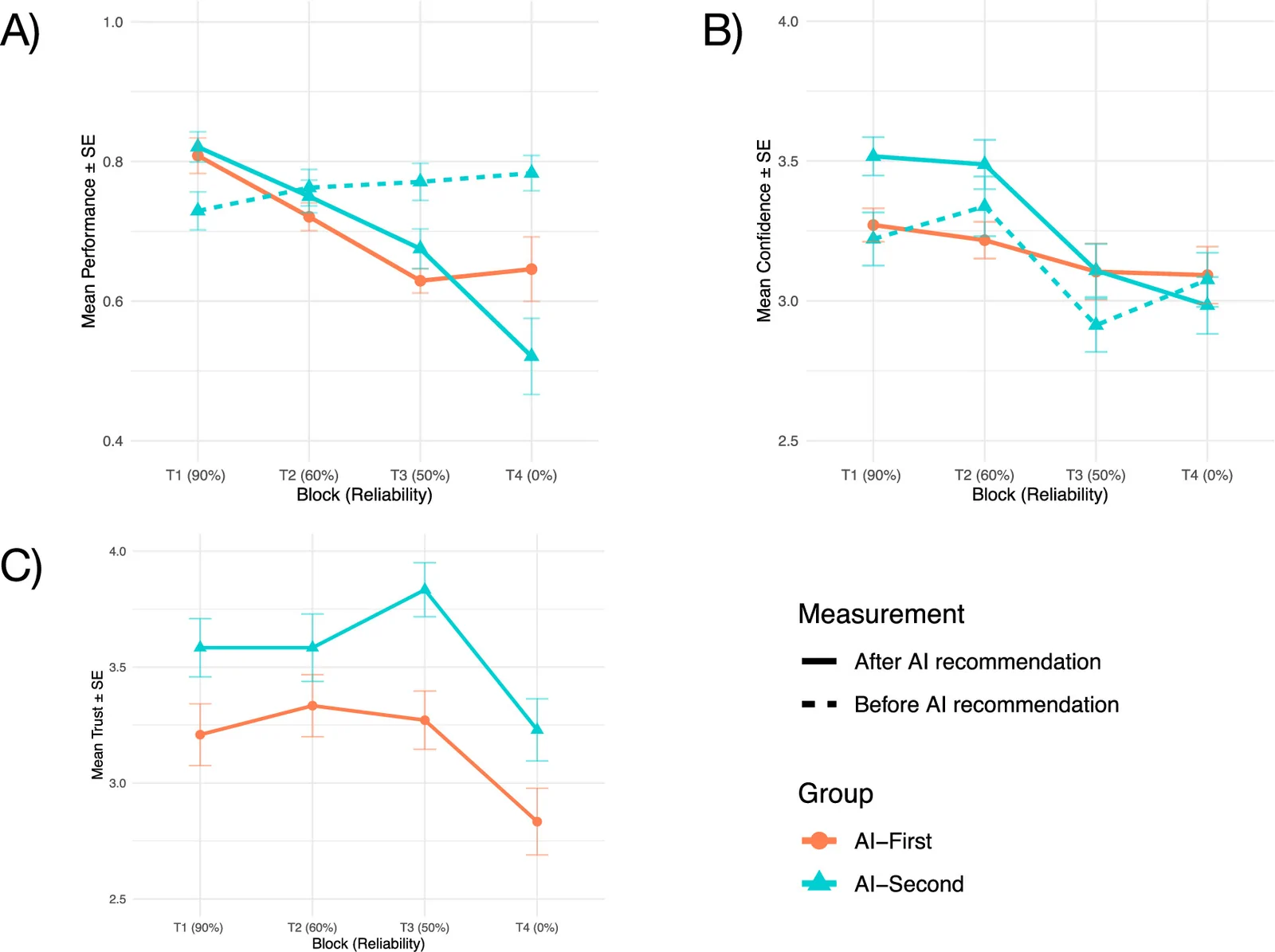
Study results. Diagnostic performance (**A**), diagnostic confidence (**B**), and trust (**C**) across four blocks with decreasing system reliability for the AI-First (orange; *n* = 24) and AI-Second (turquoise; *n* = 24) groups. Dashed turquoise lines indicate participants’ unassisted performance and confidence prior to receiving the AI recommendations (AI-Second condition only). Error bars indicate standard errors.

Figure 1 displays participants’ performance, confidence, and trust scores. Table 1 displays ANOVA results. As predicted, the AI-assisted diagnostic performance declined alongside system reliability from *M*_T1_ = 81.45% (*SD*_T1_ = 11.48%) to *M*_T4_ = 58.33% (*SD*_T4_ = 25.29%). The AI-assisted performance at T1 was lower than the AI-system reliability (90%), whereas in T2-T4 it was higher than the AI-system reliability, but lower than human unassisted performance. Overall, cognitive forcing did not improve diagnostic performance: The Group×Block interaction for performance was significant, but contrary to our hypothesis, at T4 the AI-First group (*M*_T4|AI-First_ = 64.58%, *SD*_T4|AI-First_ = 22.65%) outperformed the AI-Second group (*M*_T4|AI-Second_ = 52.08%, *SD*_T4|AI-Second_ = 26.70%).

**Table 1:** ANOVA results, i.e., main effects of block (with varying reliability) and group, as well as their interaction for diagnostic performance, diagnostic confidence, and trust

Variable	Main Effect Block (Reliability)				Main Effect Group				Block × Group Interaction			
	*F*	df	*p*	η_G_² / ηp²	*F*	df	*p*	η_G_² / ηp²	*F*	df	*p*	η_G_² / ηp²
*Performance after*	21.36	2.29, 105.19†	**<0.001**†	0.23/0. 30	0.13	1, 46	0.716	>0.01/>0.01	3.25	2.29, 105.19†	**0.037**†	0.05/0.07
*Confidence after*	24.70	3, 138	**<0.001**	0.12/0. 35	0.95	1, 46	0.336	0.02 /0. 02	6.78	3, 138	**<0.001**	0.04/0.13
*Trust*	10.83	3, 138	**<0.001**	0.06/0. 19	3.90	1, 46	0.054	0.06 /0.08	0.86	3, 138	0.464	0.01/0.02
*Performance before*	0.90	3, 69	0.444	0.03/0.04	–	–	–		–	–	–	–
*Confidence before*	27.91	3, 69	**<0.001**	0.10/0.0 55	–	–	–		–	–	–	–

Performance and confidence before refer to the baseline measured only in the AI-Second condition.

The variables Performance after, Confidence after, and Trust were assessed in both groups (*n* = 48). The variables *Performance before* and *Confidence before* refer to the unassisted baseline assessment in the AI-Second group (*n* = 24). Therefore, no between-group effects and interactions can be calculated. Significant *p*-values (<0.05) are highlighted in **bold**. Effect sizes are reported as partial eta squared (η_p_^2^) and generalized eta squared (η_G_^2^). †-symbols indicate that Greenhouse Geisser corrected df and p-values are reported due to a violation of the sphericity assumption.

In both groups, participants’ confidence after the AI recommendation dropped significantly across blocks from *M*_T1_ = 3.39 (*SD*_T1_ = 0.34) to *M*_T4_ = 3.04 (*SD*_T4_ = 0.50); and we additionally found a significant Group×Block interaction. In the AI-Second group, confidence before the AI recommendation declined across blocks, although unassisted performance did not decline (dashed lines in Fig. 1A, B and Table 1). Trust declined in both groups from *M*_T1_ = 3.40 (*SD*_T1_ = 0.91) to *M*_T4_ = 3.03 (*SD*_T4_ = 0.98). There were no significant group or interaction effects for trust. After the experiment, only 12.5% (three per group) reported noticing declining system reliability, and even when explicitly asked for changes in the system, only 37.5% (AI-First: 41.7%; AI-Second: 33.3%) reported noticing reduced reliability.

Our study examined whether physicians can detect AI malfunctions and intervene effectively, and whether cognitive forcing supports these central goals of human oversight. It revealed three important insights. First, in the high-reliability block (T1), combined human-AI performance was lower than system reliability (cf. ref. ^4^). Second, when system reliability declined, combined human-AI performance was higher than system reliability, indicating physicians’ partial ability to detect and override inaccurate AI recommendations. However, under low system reliability, combined human-AI performance also declined below the unassisted human baseline. Only a minority of participants reported noticing system malfunctions. Third, cognitive forcing did not improve human-AI performance. Together with prior evidence on erroneous AI support in medical decision-making^10,16^, these findings challenge the assumption that physicians can reliably detect and correct erroneous AI outputs, and they question the supporting effect of cognitive forcing.

Automation bias^6^ may have been one reason for physicians adopting inaccurate system recommendations. However, to understand why physicians did not sufficiently override the AI even after forming an independent assessment, the observed relationship between diagnostic confidence and trust in AI^11,17^ may provide important insights. While baseline performance remained stable in the AI-Second group (dashed line in Fig. 1A), initial diagnostic confidence declined faster (T1 > T3) than trust in the AI (T1 > T4) under decreasing reliability. The AI’s reported 96% accuracy may have fostered overestimations of system reliability^18^ and increased trust and reliance^11^, leading physicians to question their own judgment rather than the AI. Even when additional signals of system malfunction were present (T4), participants did not reliably override the AI system. These exploratory findings tentatively suggest that AI outputs may possess high perceived authority, even when physicians independently process diagnostic information before receiving AI recommendations^5,6,19^. However, these interpretations require further validation.

Several aspects of our experimental setup limit generalizability of our results. Although physician experience did not affect our results, 38% of participants were physicians in training. Additionally, the study was conducted in a controlled lab environment focusing on an isolated diagnostic subtask without physician-patient interaction or access to additional clinical information (e.g., vital signs, general patient presentation). Our manipulation of system malfunction included a rapid continuous decline in system reliability resulting in 0% accuracy and desynchronized auditory and visual cues. Consequently, fluctuations in reliability were not captured, order effects cannot be excluded, and we cannot conclusively tell which aspect of the system malfunction (i.e., reliability decline or desynchronization) mainly yielded the results pattern at T4. In clinical practice, system malfunctions might be more heterogeneous, e.g., more subtle or fluctuating, and real-world factors, such as fatigue, competing demands, and time pressure, likely make oversight of AI even more challenging^5,20^. Furthermore, prolonged reliance on highly accurate AI systems may increase trust^5^ while eroding manual diagnostic skills and reducing diagnostic confidence^21^.

For future research and practice, ensuring effective human oversight^2,22^ requires clearly defined success criteria. Human oversight may be considered successful if human-AI performance either (1) exceeds AI performance, or (2) does not fall below unassisted human performance. Regarding (1), our data suggest that physicians may struggle when interacting with highly reliable systems, where human involvement can reduce AI-only performance (T1, cf. refs. ^4,20^). Regarding (2), although physicians mitigated some negative effects of declining system reliability, low-reliability AI can deteriorate outcomes below unassisted human performance (T2-T4, cf. ref. ^4^).

In our experiment, cognitive forcing did not improve human oversight effectiveness. Nevertheless, further research in realistic settings is necessary to better understand cognitive-forcing effects, particularly under conditions of multiple task demands, access to additional patient information, other AI use cases (e.g., image classification, treatment planning), or dedicated training in which oversight roles are clearly specified. Beyond performance, additional outcomes should be considered. For instance, outsourcing tasks to AI may contribute to deskilling^21^, whereas requiring independent assessments (as in the cognitive forcing condition) may help preserve diagnostic skills, despite increasing diagnostic time. These trade-offs should be considered when designing human oversight workflows.

Since our results suggest that AI-system malfunctions may be difficult for physicians to detect, effective human oversight may require additional action at the system and organizational levels^22^. AI systems should provide relevant cues to facilitate monitoring and error detection, such as uncertainty indicators or explanations^23^. At the organizational level, accountability may need to be shared with stakeholders such as AI deployers (e.g., hospitals) and providers (e.g., developers)^24^. Compared to physicians who treat patients under time pressure, these actors may be better positioned to detect system biases and malfunctions at scale by using monitoring tools that can track shifts in prevalence and subgroup distributions. Combined with physicians’ informed clinical input and transparent system design, such structures may form a more robust safety net against AI malfunctions.

To conclude, our study provides initial evidence that human oversight alone may be insufficient to reliably detect system malfunctions in AI-assisted cardiac auscultation. Despite their expertise, physicians often failed to detect declining system reliability, even with additional evidence for system malfunctions (i.e., desynchronization) and when employing cognitive forcing techniques. Starting with a clear definition of oversight goals, future research should examine how physician-, system-, and organizational-level factors contribute to effective oversight in clinical workflows.

## Methods

### Ethics and inclusion

The study was conducted in accordance with the Declaration of Helsinki. The medical ethics board of the Philipps-Universität Marburg approved the study prior to conducting it under the reference approval number 23–69 BO. All participants provided informed consent before participation. Data collection was anonymous and adhered to GDPR guidelines to protect confidentiality. Our sample included medical professionals across different experience levels; it was limited to German-speaking participants. All participants were debriefed after the study and had the opportunity to sign up to receive study results.

### Participants

This study was preregistered on aspredicted.org (#148,334; Clinical trial number: not applicable; https://aspredicted.org/78v7-jpsp.pdf). The sample size was determined a priori via a power analysis using G*Power^25^ for the statistical methods employed. The required sample size (*N* = 48) was calculated to detect an effect size of η_p_² = 0.03 in a mixed-ANOVA interaction with 1-β = 0.80 and α = 0.05.

Between October 2023 and May 2024, we recruited 48 physicians (*M*_age_ = 35.48 years, *SD* = 16.04; 64.6% male, 35.4% female) through direct outreach, posters, and snowball sampling. All participants completed the study voluntarily. No data were excluded from the analyses. Participants were randomly assigned to either the AI-First group (*n* = 24) or the AI-Second group (*n* = 24). The sample consisted of 37.50% medical students in their final year, 39.58% resident physicians (i.e., physicians licensed to practice under supervision of experienced physicians, usually in their first 6 years after graduation), and 22.92% experienced physicians (including specialists, senior physicians, private practitioners, and chief doctors). Specialties varied considerably (general & internal medicine [18%], anesthesiology [14%], cardiology [11%], others [15%]). Mean professional experience across the full sample was *M* = 8.19 years, *SD* = 14.33. Mean professional experience of practicing physicians (excluding *n* = 18 medical students in their final year) was *M* = 12.93 years, *SD* = 16.43. 45.83% of the sample reported no professional experience. Overall, self-assessed auscultation ability (from 1 = “very bad” to 5 = “very good”) was at *M* = 3.00, *SD* = 0.77, and 16.67% reported prior experience with digital auscultation. Their affinity for technology interaction^26^ was at a medium level (*M* = 3.92, *SD* = 0.99). Experimental groups did not differ significantly in any of these demographic variables (Table S14).

### Experimental design and procedure

We employed a 2 × 4 mixed factorial design, experimentally manipulating the timing of the AI recommendation (between-subjects: AI-First vs. AI-Second), and declining system reliability (within-subjects: 90% vs. 60% vs. 50% vs. 0%).

Experiments were conducted in a quiet environment, using standardized Sony WH-1000XM4 headphones with replaceable covers for hygiene. To facilitate participation, data collection was conducted across multiple locations while maintaining consistent sound conditions. Demographic information and post-experimental measures were collected using an online questionnaire administered on a tablet device via the survey platform “SoSci Survey”. Participants completed the experiment on a 13-inch MacBook Pro with standardized instructions and no additional experimenter interaction. The implementation of the experiment was mainly based on the *Pygame* (2.5.2) library.

Note that, after the computer experiment, participants completed an additional survey in which, for example, their affinity for technological interaction and acceptance of technology were assessed. Due to space restrictions, we focus here on the data collected in the computer experiment and do not provide detailed information on the survey data. All items of the post-experimental survey (Tables S2 and S3) and a correlational table (Table S15) are available in the online supplement, and complete data is provided at https://osf.io/836ny/?view_only=3a1631a54edd489197a8b3b9e4322fa0.

Participants were randomly assigned to one of the two experimental groups: AI-First or AI-Second (indicating the timing of receiving the AI recommendation, where AI-Second entails cognitive forcing, in which participants first have to come up with their own classification before receiving system advice). Initially, all participants provided demographic data. Afterwards, to provide a realistic setting while remaining under full experimental control, participants were introduced to a telemedical auscultation scenario on the computer, in which they were asked to review digitally recorded heart sounds and classify them as “normal” or “abnormal”. Note that, to control for unwanted variations in the participants’ assessment process of the heart sounds and to align the assessment with the ground truth from the dataset we used^27,28^, the classification of “abnormal” was described as excluding rhythm disturbances and tachycardias/bradycardias, but including pathological flow noises or heart murmurs. Participants were subsequently informed about the AI system that was provided to support their decision-making. First, they received an explanation of the two types of visualizations provided by the system: Phonocardiogram and Mel-spectrogram (see Fig. S5 in the online supplement). Second, they received information about the training data (i.e., 8425 files from 1500 individuals between the ages of 0 and 21), and the AI’s reliability in the validation (i.e., 96% accuracy with 97% sensitivity and 89% specificity). This reliability information was based on published AI cardiac auscultation performance data^29^. All participants were informed about the experimental procedure in their respective groups and were unaware of the other group. The complete original and translated instructions are available in the online supplement (Figs. S1–S12). Before starting, participants conducted a practice trial, which they could repeat as often as they wanted.

The main experiment consisted of 40 trials in blocks of 10 with decreasing system reliability (90%, 60%, 50%, and 0%; see subsequent section for more information on the audio files). Note, in block 4, we deliberately added an audio-visual desynchrony, i.e., the audio and visual components were sampled from different recordings. The blocks were always presented in the same order to simulate a degrading system performance over time. The trials within the blocks were presented in a pseudorandomized order; each block contained the same ten trials, but their order was randomized to control for order effects. After completing two blocks (20 trials), participants had the opportunity to take a break and resume the task at their own pace.

**Fig. 2:**
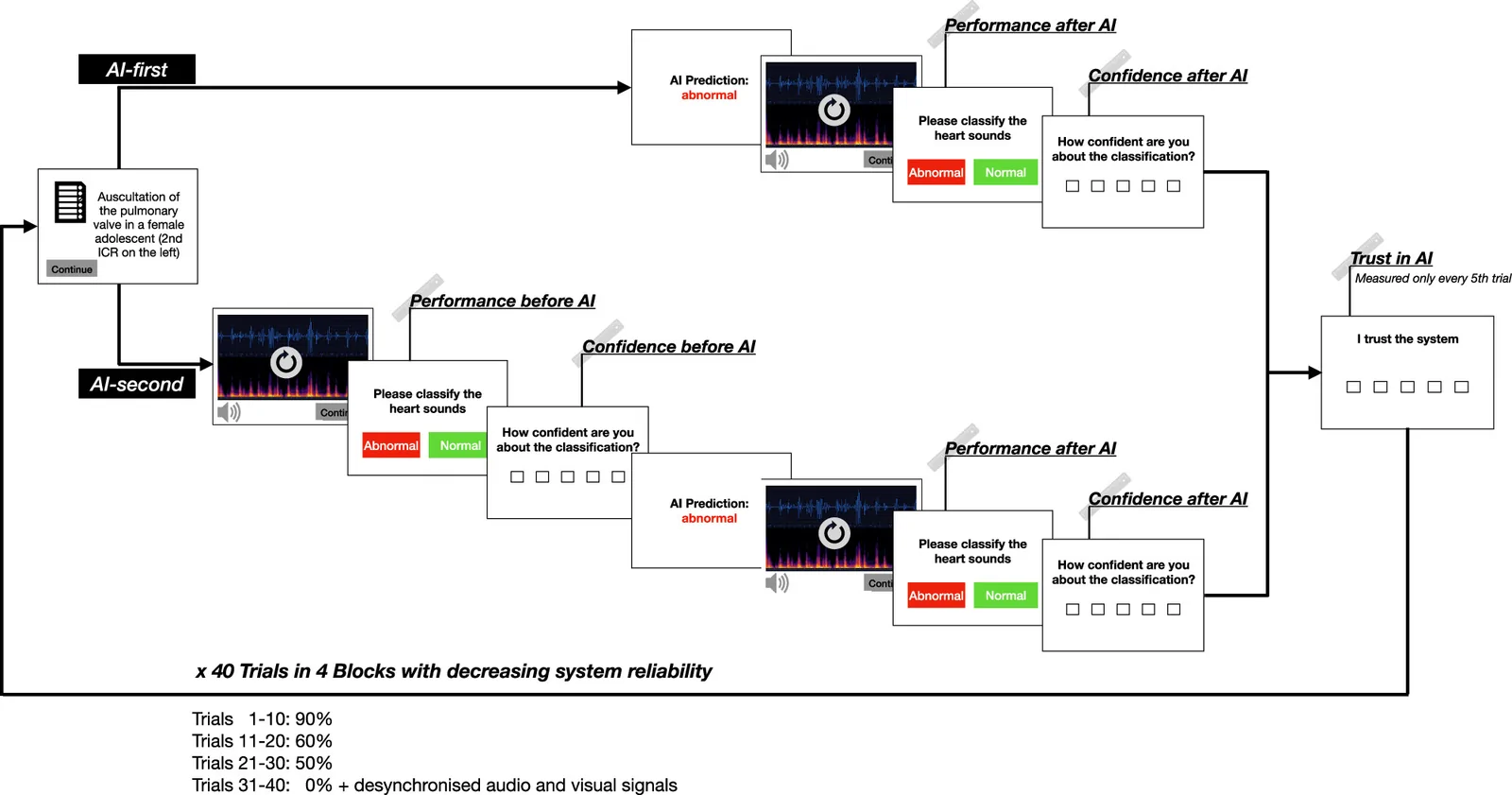
Experimental design of the current study. This figure depicts one trial run of the 40 trials for each group. A sample trial in the AI-First group is presented at the top, and a sample trial in the AI-Second group is presented at the bottom. Note that participants in both groups could replay the auscultation file as many times as they wanted, as indicated by the replay icon.

Each trial followed a structured sequence depending on the experimental group (Fig. 2). Initially, all participants viewed an on-screen patient vignette (containing information about the patient’s age cohort, sex, and the auscultation point as described in the data set^27^). Participants in the AI-First group then received the AI recommendation (“normal” or “abnormal”) directly before the presentation of the heart sound recording that included the heart sound and its visualization as a phonocardiogram and a Mel-spectrogram. Participants could replay the video as many times as they wanted. Then, they were asked to classify the heart sound as normal or abnormal, and to report their diagnostic confidence. After each fifth trial, they were also asked how much they trusted the AI system.

Participants in the AI-Second group first listened to the recording, which they could replay as many times as they wanted. Then, they classified the heart sound as either “normal” or “abnormal” and reported their diagnostic confidence. Note that, as the audio and visualization of this initial assessment were always identical to the audio and visualization after receiving an AI recommendation, this assessment can be interpreted as an unassisted human performance baseline. Afterwards, the procedure was similar to that of the AI-First group. Participants received the AI recommendation and could replay the recording as many times as they wanted. Then, they were asked to classify the heart sound, provide their diagnostic confidence, and, after each fifth trial, rate their trust in the system. After completing the computer-based experiment, participants resumed the initial online survey on a tablet device, where, among others, qualitative responses were collected.

### Materials

The main dependent variables reported here were assessed as follows: *Diagnostic performance* was calculated as the proportion of participants’ correct classifications of heart sounds (normal vs. abnormal) relative to the gold-standard diagnoses from the dataset^28^. *Diagnostic confidence* was assessed in each trial and every time when participants provided a decision (i.e., before and after receiving the AI recommendation) using the item^30^: “How confident are you in your classification?” and responded on a 5-point rating scale (1 = “Not confident at all – I am guessing”, 2 = “Not very confident”, 3 = “Somewhat confident”, 4 = “Fairly confident”, 5 = “Absolutely confident”). *Trust in system support* was assessed after every fifth trial. Participants rated the statement “I trust the system” on a 5-point Likert scale (1 = “Strongly disagree”, 2 = “Disagree”, 3 = “Neutral”, 4 = “Agree”, 5 = “Strongly agree”)^31^. All items used in this study can be found in Tables S1–S3 of the online supplement, which provides the original German versions alongside their English translations.

To prepare the audio stimuli for this study, initially 40 heart sound recordings were selected from the training data (version 1.0.2) within the open CirCor dataset^27,28^, which comprised 3163 recordings from 942 individuals. The files were visualized using an AI tool, but the AI recommendations were manually edited to fit our intended study design. Each block contained 50% normal and 50% abnormal recordings to avoid rater biases^32^. We actively manipulated the error rates of the AI to increase the false-positive rate. This was done to better reflect real recommender systems, which also tend to provide more false positives^33^, and because the system information that we provided to participants indicated that the system was more likely to produce false-positive recommendations (i.e., that the system has a higher sensitivity than specificity). Thus, we had one false positive in block 1, three false positives and one false negative in block 2, and three false positives and two false negatives in block 3. Block 4 consisted of five false positives and five false negatives with audio-visual asynchrony. The audio files were piloted to achieve similar difficulty across all blocks. A detailed description of selection criteria, stimuli preparation, and the pilot study is available in the online supplement.

### Data treatment and analyses

Data were analyzed using RStudio (version 2023.09.1 + 494). Independent-samples ANOVAs were used for comparisons between groups, while repeated-measures ANOVAs were employed for within-group comparisons. To analyze interaction effects between the within-subject factor *block* and the between-subject factor *group*, mixed-design ANOVAs were conducted. Violations of assumptions were assessed using mainly visual residual inspection complemented by Mauchly's test (for violation of sphericity), Shapiro-Wilk test (for violation of normality), and Levene test (for violation of homogeneity of variances). In case of violated assumptions of sphericity, Greenhouse-Geisser correction was applied. Assumptions of normality and homogeneity of variances were not severely violated, and hence no correction was applied. Effect sizes are reported as partial eta squared (η_p_^2^)^34^, and generalized eta squared (η_G_^2^) to increase comparability with other experimental designs^35^.

As a sensitivity analysis, and to control for potential influencing nested variables, we additionally assessed interaction effects with hierarchical linear models with participant-ID as the random factor to account for within-participant variance. Participants’ classification was evaluated using a generalized linear mixed model (GLMM) for binary outcomes with a logit link function. The model included random intercepts for participants, along with fixed effects for block (90%-0%) and group (AI-First vs. AI-Second). We fitted additional models controlling for participants’ work experience and self-assessed auscultation skill. The re-transformed effect estimate for the classification variable is reported as an odds ratio with the corresponding 95% confidence interval (CI) as the main confirmatory result. The significance threshold was set at α = .05, and two-tailed *p*-values are reported (i.e., non-halved *p*-values). Sensitivity analyses led to the same conclusions as our main analyses, even when controlling for participants’ work experience or their self-assessed auscultation skill (see Tables S4–S10 in the online supplement).

The qualitative coding of the two open-ended questions was conducted independently by two researchers (C.T. & N.S.). Initial interrater reliability was moderate and ranged from κ = 0.54 to κ = 0.56 (*p* < 0.001) for the two qualitative questions. Deviations were discussed and resolved for further analyses.

## Supplementary information

Supplement_Human_Oversight_Medical_AI.

## Data Availability

Full data is available at: https://osf.io/836ny/overview?view_only=3a1631a54edd489197a8b3b9e4322fa0.

## Code availability

Data was analyzed using RStudio (version 2023.09.1 + 494). Analysis code is available at: https://osf.io/836ny/?view_only=3a1631a54edd489197a8b3b9e4322fa0. The implementation of the experiment was mainly based on the *pygame* (2.5.2) library. The code for running the experiment is available at: https://github.com/IKI-Lab/human_oversight_auscultation.

## References

[1] Aboy, M., Minssen, T. & Vayena, E. Navigating the EU AI Act: Implications for regulated digital medical products. *Npj Digit. Med.* **7**, 1–6 (2024). 10.1038/s41746-024-01232-3 PMC11379845 39242831

[2] Green, B. The flaws of policies requiring human oversight of government algorithms. *Comput. Law Secur. Rev.* **45**, 105681 (2022).

[3] Tag, B. et al. DDoD: Dual denial of decision attacks on human-AI teams. *IEEE Pervas. Comput* **22**, 77–84 (2023).

[4] Vaccaro, M., Almaatouq, A. & Malone, T. When combinations of humans and AI are useful: A systematic review and meta-analysis. *Nat. Hum. Behav.* **8**, 2293–2303 (2024). 10.1038/s41562-024-02024-1 PMC11659167 39468277

[5] Parasuraman, R. & Manzey, D. H. Complacency and bias in human use of automation: An attentional integration. *Hum. Factors* **52**, 381–410 (2010). 10.1177/0018720810376055 21077562

[6] Mosier, K. L. & Skitka, L. J. Human Decision Makers and Automated Decision Aids: Made for Each Other? in *Automation and Human Performance* (CRC Press, 1996).

[7] Tversky, A. & Kahneman, D. Judgment under uncertainty: Heuristics and biases. *Science* **185**, 1124–1131 (1974). 10.1126/science.185.4157.1124 17835457

[8] Bach, A. K. P., Nørgaard, T. M., Brok, J. C. & van Berkel, N. ‘If I had all the time in the world’: Ophthalmologists’ perceptions of anchoring bias mitigation in clinical AI support. *Proceedings of the 2023 CHI Conference on Human Factors in Computing Systems*. 1–14 10.1145/3544548.3581513 (2023).

[9] Jussupow, E., Spohrer, K., Heinzl, A. & Gawlitza, J. Augmenting medical diagnosis decisions? An investigation into physicians’ decision-making process with artificial intelligence. *Inf. Syst. Res.* **32**, 713–735 (2021).

[10] Biro, J. M. et al. Opportunities and risks of artificial intelligence in patient portal messaging in primary care. *Npj Digit. Med.* **8**, 222 (2025). 10.1038/s41746-025-01586-2 PMC12022076 40275104

[11] Parasuraman, R. & Riley, V. Humans and automation: Use, misuse, disuse, abuse. *Hum. Factors* **39**, 230–253 (1997).

[12] Buçinca, Z., Malaya, M. B. & Gajos, K. Z. To trust or to think: Cognitive forcing functions can reduce overreliance on AI in AI-assisted decision-making. *Proc. ACM Hum. -Comput. Interact.* **5**, 1–21 (2021).

[13] de Jong, S. et al. Cognitive forcing for better decision-making: Reducing overreliance on AI systems through partial explanations. *Proc. ACM Hum. -Comput. Interact.* **9**, 1–30 (2025).

[14] Rittenberg, B. S. P., Holland, C. W., Barnhart, G. E., Gaudreau, S. M. & Neyedli, H. F. Trust with increasing and decreasing reliability. *Hum. Factors* **66**, 2569–2589 (2024). 10.1177/00187208241228636 PMC11487872 38445652

[15] Langer, M., Baum, K. & Schlicker, N. Effective human oversight of AI-based systems: A signal detection perspective on the detection of inaccurate and unfair outputs. *Minds Mach*. **35**, 1–30 (2024).

[16] Ryan, K., Yang, H.-J., Kim, B. & Kim, J. P. Assessing the impact of AI on physician decision-making for mental health treatment in primary care. *Npj Ment. Health Res.* **4**, 16 (2025). 10.1038/s44184-025-00124-y PMC12065820 40348864

[17] de Vries, P., Midden, C. & Bouwhuis, D. The effects of errors on system trust, self-confidence, and the allocation of control in route planning. *Int. J. Hum. -Comput. Stud.* **58**, 719–735 (2003).

[18] Schlicker, N. et al. How do we assess the trustworthiness of AI? Introducing the trustworthiness assessment model (TrAM). *Comput. Hum. Behav.* **170**, 108671 (2025).

[19] Jakesch, M., Bhat, A., Buschek, D., Zalmanson, L. & Naaman, M. Co-writing with opinionated language models affects users’ views. *Proceedings of the 2023 CHI Conference on Human Factors in Computing Systems*. 1–15 (2023) 10.1145/3544548.3581196.

[20] Rieger, T. & Manzey, D. Human performance consequences of automated decision aids: The impact of time pressure. *Hum. Factors* **64**, 617–634 (2022). 10.1177/0018720820965019 33111557

[21] Budzyń, K. et al. Endoscopist deskilling risk after exposure to artificial intelligence in colonoscopy: A multicentre, observational study. *Lancet Gastroenterol. Hepatol.* **10**, 896–903 (2025). 10.1016/S2468-1253(25)00133-5 40816301

[22] Sterz, S. et al. On the quest for effectiveness in human oversight: Interdisciplinary perspectives. *Proceedings of the 2024 ACM Conference on Fairness, Accountability, and Transparency.* 2495–2507 10.1145/3630106.3659051 (2024).

[23] Lim, B. Y., Cahaly, J. P., Sng, C. Y. F. & Chew, A. Diagrammatization and abduction to improve AI interpretability with domain-aligned explanations for medical diagnosis. *Proceedings of the 2023 CHI Conference on Human Factors in Computing Systems.* 1–25 (2025) 10.1145/3706598.3714058.

[24] Grote, G., Parker, S. K. & Crowston, K. Taming artificial intelligence: A theory of control-accountability alignment among AI developers and users. *Acad. Manage. Rev*. Advance Online Publication (2024).

[25] Faul, F., Erdfelder, E., Lang, A.-G. & Buchner, A. G. Power 3: A flexible statistical power analysis program for the social, behavioral, and biomedical sciences. *Behav. Res. Methods* **39**, 175–191 (2007). 10.3758/bf03193146 17695343

[26] Franke, T., Attig, C. & Wessel, D. A personal resource for technology interaction: Development and validation of the affinity for technology interaction (ATI) scale. *Int. J. Hum.–Comput. Interact.* **35**, 456–467 (2019).

[27] Oliveira, J. et al. The CirCor DigiScope Phonocardiogram Dataset. PhysioNet 10.13026/tshs-mw03. (2022).

[28] Oliveira, J. et al. The CirCor DigiScope Dataset: From murmur detection to murmur classification. *IEEE J. Biomed. Health Inform.* **26**, 2524–2535 (2022). 10.1109/JBHI.2021.3137048 PMC9253493 34932490

[29] Lv, J. et al. Artificial intelligence-assisted auscultation in detecting congenital heart disease. *Eur. Heart J. - Digit. Health* **2**, 119–124 (2021). 10.1093/ehjdh/ztaa017 PMC9708038 36711176

[30] Wiczorek, R. & Meyer, J. Effects of trust, self-confidence, and feedback on the use of decision automation. *Front. Psychol*. **10**, 1–12 (2019). 10.3389/fpsyg.2019.00519 PMC6423180 30915005

[31] Körber, M. Theoretical considerations and development of a questionnaire to measure trust in automation. *Proc. 20th Congr. Int. Ergon. Assoc. IEA 2018* 13–30 (2019) 10.1007/978-3-319-96074-6_2.

[32] Stanislaw, H. & Todorov, N. Calculation of signal detection theory measures. *Behav. Res. Methods Instrum. Comput.* **31**, 137–149 (1999). 10.3758/bf03207704 10495845

[33] Fernandes, C. O., Miles, S., Lucena, C. J. P. D. & Cowan, D. Artificial intelligence technologies for coping with alarm fatigue in hospital environments because of sensory overload: Algorithm development and validation. *J. Med. Internet Res.* **21**, e15406 (2019). 10.2196/15406 PMC6904899 31769762

[34] Cohen, J. *Statistical Power Analysis for the Behavioral Sciences*. (Routledge, 2013).

[35] Lakens, D. Calculating and reporting effect sizes to facilitate cumulative science: A practical primer for t-tests and ANOVAs. *Front. Psychol.* **4**, 863 (2013). 10.3389/fpsyg.2013.00863 PMC3840331 24324449

