# Supplementary material for: Challenges for human oversight in detecting system malfunctions in AI-assisted medical diagnostics
Source: NPJ Digit Med. 2026 Nov 13;9:889. doi: 10.1038/s41746-026-03410-x (PMC13768336; doi:10.1038/s41746-026-03410-x)
Supplement: Supplementary file 1 [file 41746_2026_3410_MOESM1_ESM.pdf]

Online Supplement

of „ Challenges for Human Oversight in Detecting System Malfunctions in AI-Assisted Medical Diagnostics"

Table of Contents

Instructions* *2

Items Used in the Study* *11

Stimulus Selection* *16

Supplementary Data Analysis* *19

Sensitivity Analysis 19

Performance 20

Confidence 23

Trust 25

Exploratory Analysis 28

Sample Characteristics 30

Correlation Table 31

References* *33

Instructions

In the following we provide additional details on the instructions participants received at the laptop during the experiment. We provide screenshots of the original experiment in chronological order to show the actual visuals and an English translation in the captions of the respective figures.

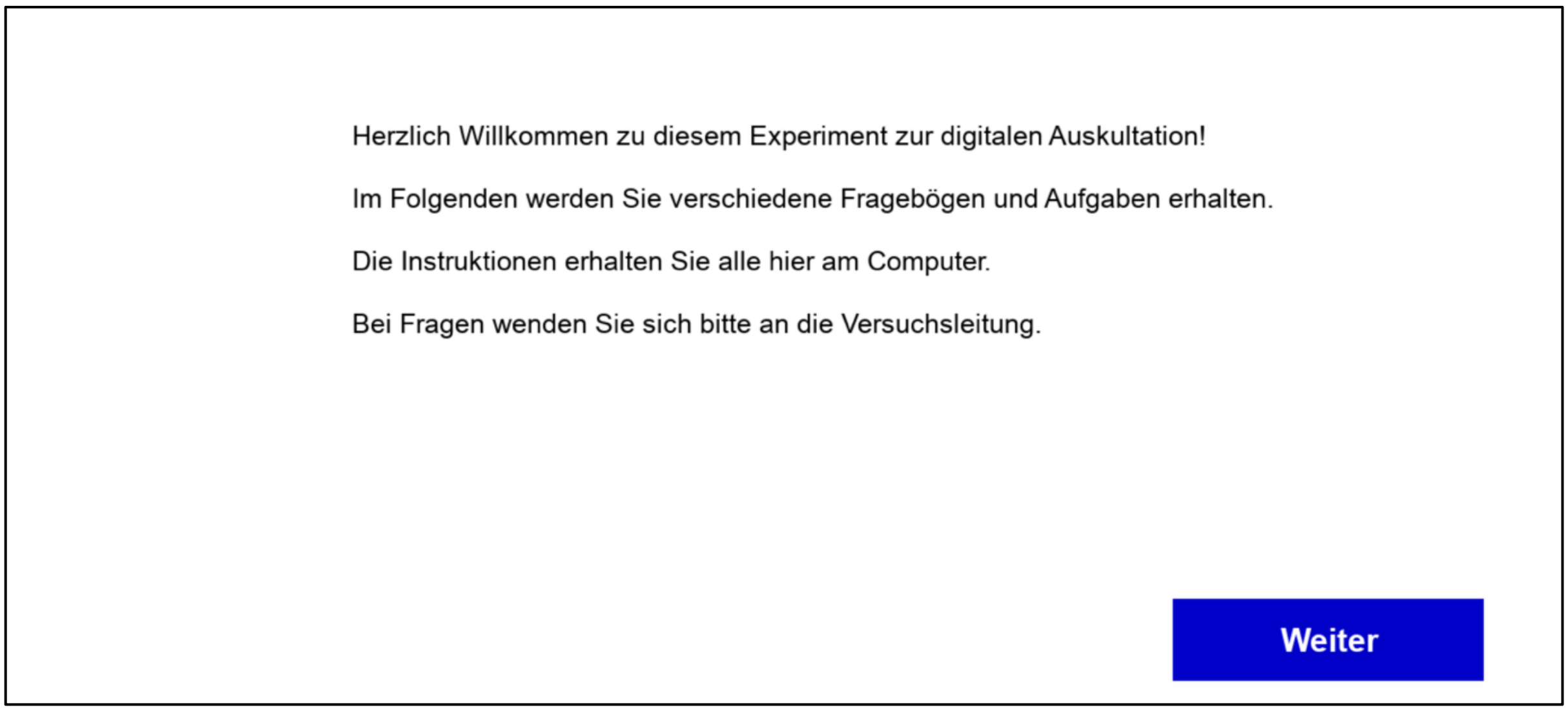

Figure S1. Welcome Screen. “Welcome to this experiment on digital auscultation! You will soon receive different questionnaires and tasks. All instructions will be provided on this computer. If you have any questions, please consult the experimenter.”

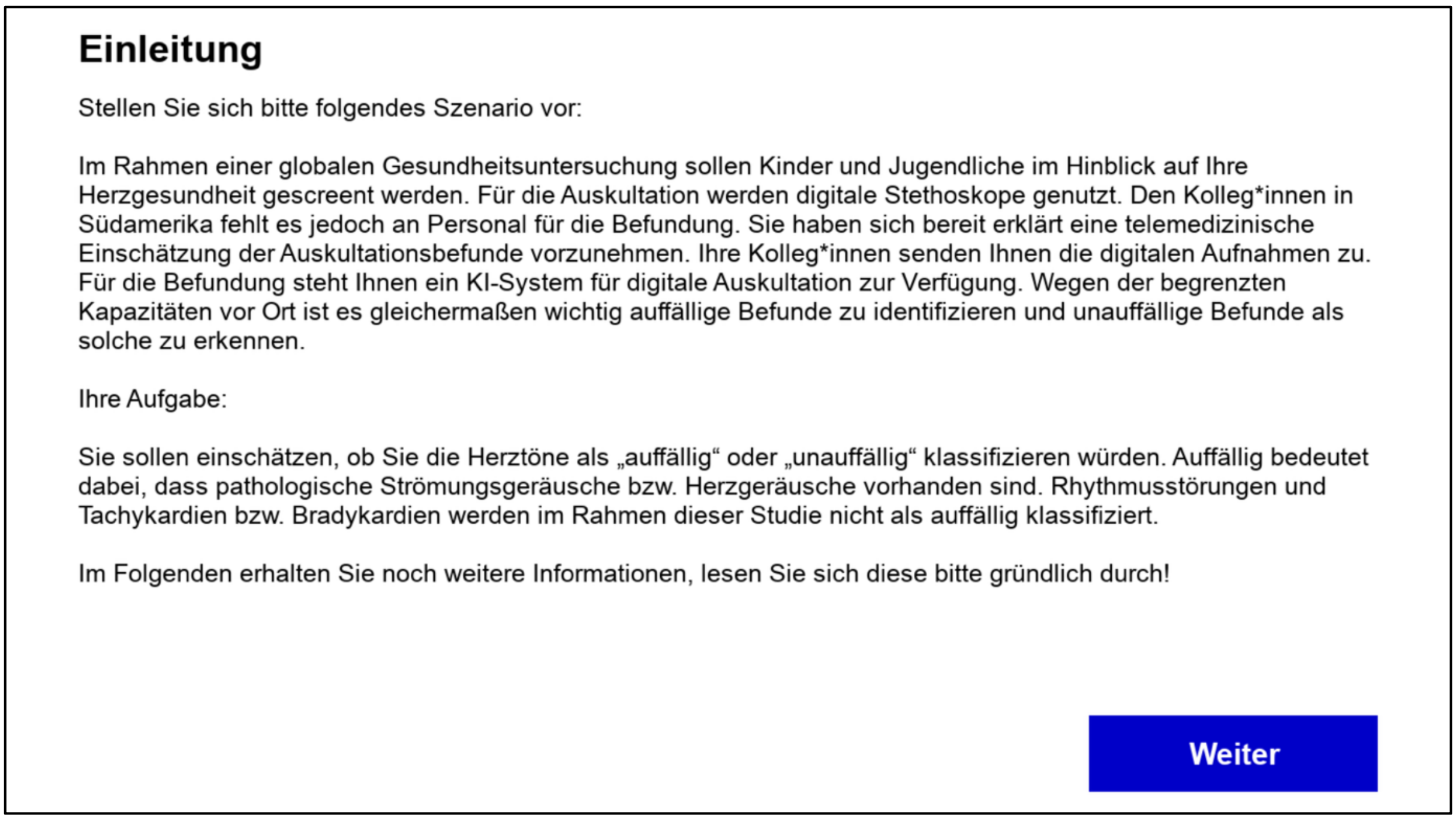

Einleitung

Stellen Sie sich bitte folgendes Szenario vor:

Im Rahmen einer globalen Gesundheitsuntersuchung sollen Kinder und Jugendliche im Hinblick auf Ihre Herzgesundheit gescreent werden. Für die Auskultation werden digitale Stethoskope genutzt. Den Kolleg*innen in Südamerika fehlt es jedoch an Personal für die Befundung. Sie haben sich bereit erklärt eine telemedizinische Einschätzung der Auskultationsbefunde vorzunehmen. Ihre Kolleg*innen senden Ihnen die digitalen Aufnahmen zu. Für die Befundung steht Ihnen ein KI-System für digitale Auskultation zur Verfügung. Wegen der begrenzten Kapazitäten vor Ort ist es gleichermaßen wichtig auffällige Befunde zu identifizieren und unauffällige Befunde als solche zu erkennen.

Ihre Aufgabe:

Sie sollen einschätzen, ob Sie die Herztöne als „auffällig“ oder „unauffällig“ klassifizieren würden. Auffällig bedeutet dabei, dass pathologische Strömungsgeräusche bzw. Herzgeräusche vorhanden sind. Rhythmusstörungen und Tachykardien bzw. Bradykardien werden im Rahmen dieser Studie nicht als auffällig klassifiziert.

Im Folgenden erhalten Sie noch weitere Informationen, lesen Sie sich diese bitte gründlich durch!

Weiter

Figure S2. Introduction. “Please imagine the following scenario: Children and adolescents are to be screened for heart health as part of a global health check-up. For the auscultation digital stethoscopes are used. The colleagues in South America are short of staff and you have therefore agreed to carry out a telemedical assessment of the auscultation findings. Your colleagues will send you the digital recordings. An AI system for digital auscultation is available for the diagnosis. Due to the limited capacities on site, it is equally important to identify abnormal findings and to recognize normal findings as such.

Your task: You are to assess whether you would categorize the heart sounds as abnormal or normal. Abnormal means that pathological flow sounds or heart murmurs are present. Rhythm disturbances and tachycardia or bradycardia are not classified as abnormal in this study. In the following you will receive additional information. Please read this information carefully.”

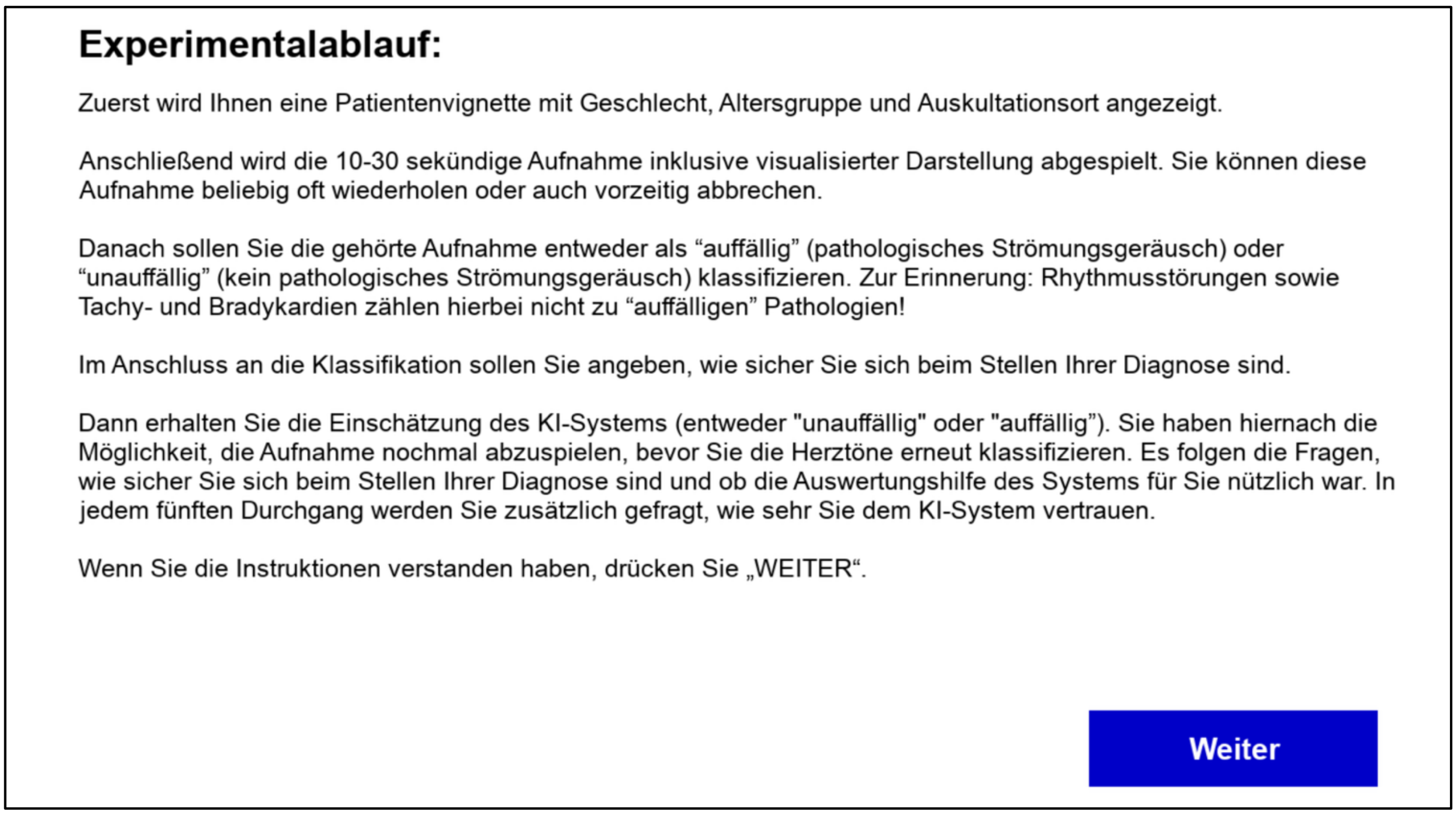

Figure S3. Experimental instruction in the *AI-Second group*. “First, you will be shown a patient vignette including gender, age cohort, and auscultation location. Next, a 10–30 second recording with visual representation will be played. You may replay the recording as many times as you like or stop it early. Afterward, you are to classify the recording you heard as either “abnormal” (pathological flow murmur) or “normal” (no pathological flow murmur). Reminder: Rhythm disorders such as tachycardia and bradycardia do not count as “abnormal” pathologies! Following the classification, you will indicate how confident you are in your diagnosis. You will then receive the AI system's assessment (either "normal" or "abnormal"). After this, you will have the option to replay the recording before classifying the heart sounds again. This will be followed by questions about how confident you were in your diagnosis and whether the AI system's evaluation assistance was useful to you. In every fifth trial, you will also be asked how much you trust the AI system. If you have understood the instructions, press ‘CONTINUE.’”

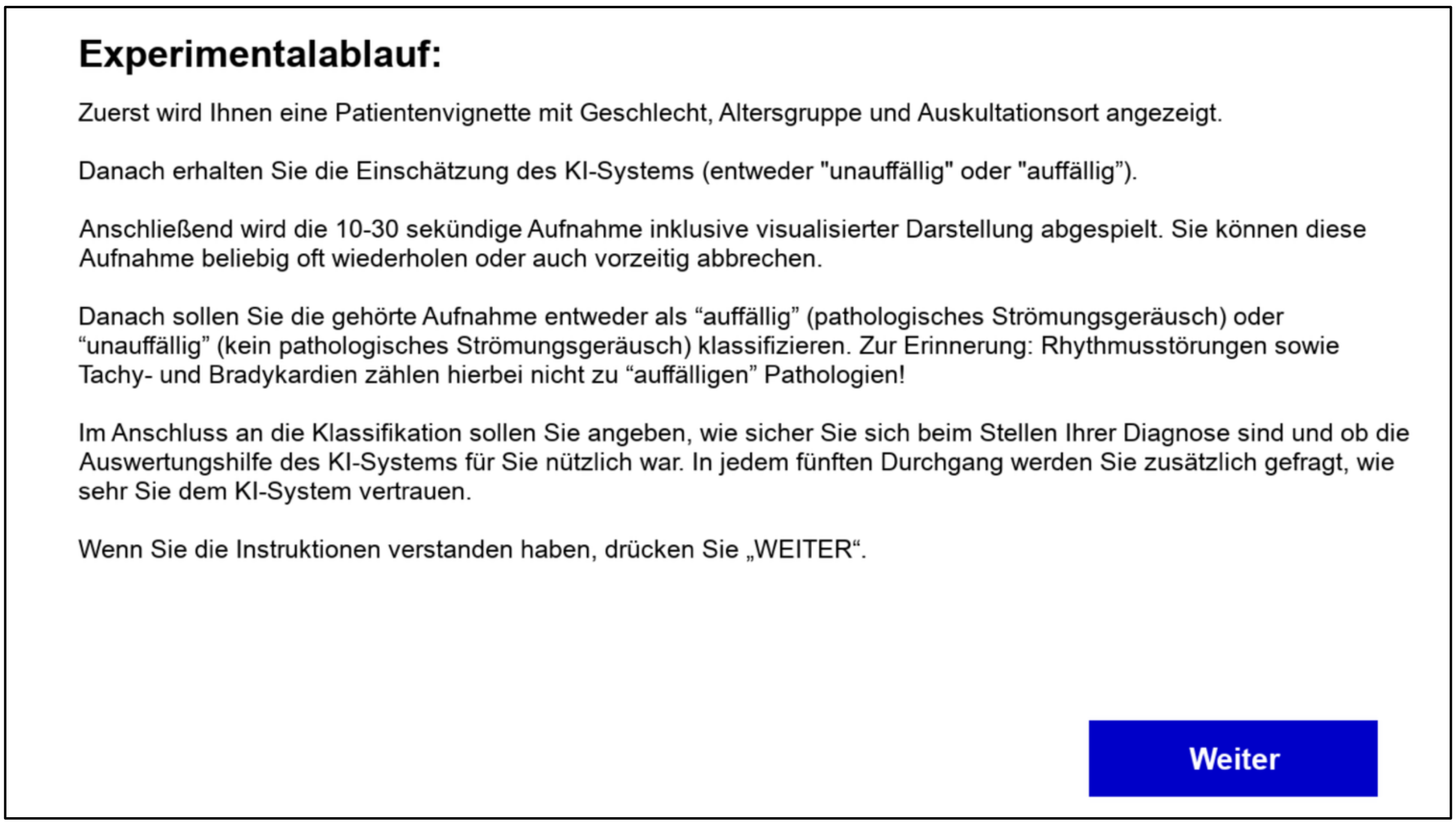

Figure S4. Experimental instruction in the *AI-First group*. "First, you will be shown a patient vignette including gender, age cohort, and auscultation location. Then you will receive the AI system's assessment (either "normal" or "abnormal"). Next, a 10–30 second recording with visual representation will be played. You may replay the recording as many times as you like or stop it early. Afterward, you are to classify the recording you heard as either "abnormal" (pathological flow murmur) or "normal" (no pathological flow murmur). Reminder: Rhythm disorders such as tachycardia and bradycardia do not count as "abnormal" pathologies! Following the classification, you will indicate how confident you are in your diagnosis and whether the AI system's analysis assistance was useful to you. After every fifth trial, you will also be asked how much you trust the AI system. If you have understood the instructions, press 'CONTINUE.'"

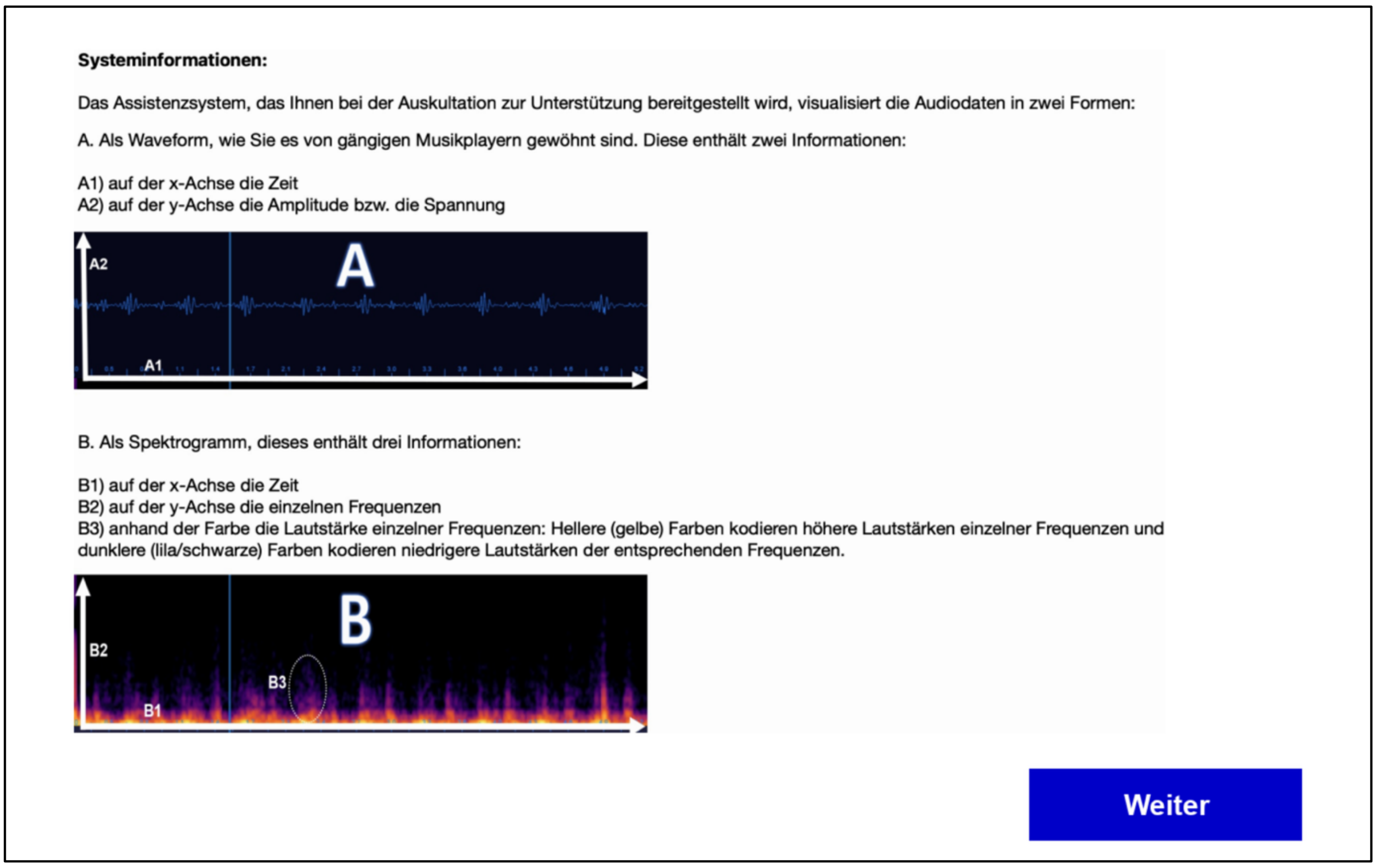

Figure S5. System information part 1. "The assistance system provided to support your auscultation process visualizes the audio data in two forms:

A. As a waveform, similar to what you're used to from common music players. It contains two pieces of information:

A1) Time on the x-axis

A2) Amplitude or voltage on the y-axis

B. As a spectrogram, which contains three pieces of information:

B1) Time on the x-axis

B2) Individual frequencies on the y-axis

B3) Volume of individual frequencies represented by color: Brighter (yellow) colors indicate higher volumes of individual frequencies, while darker (purple/black) colors indicate lower volumes at those frequencies.

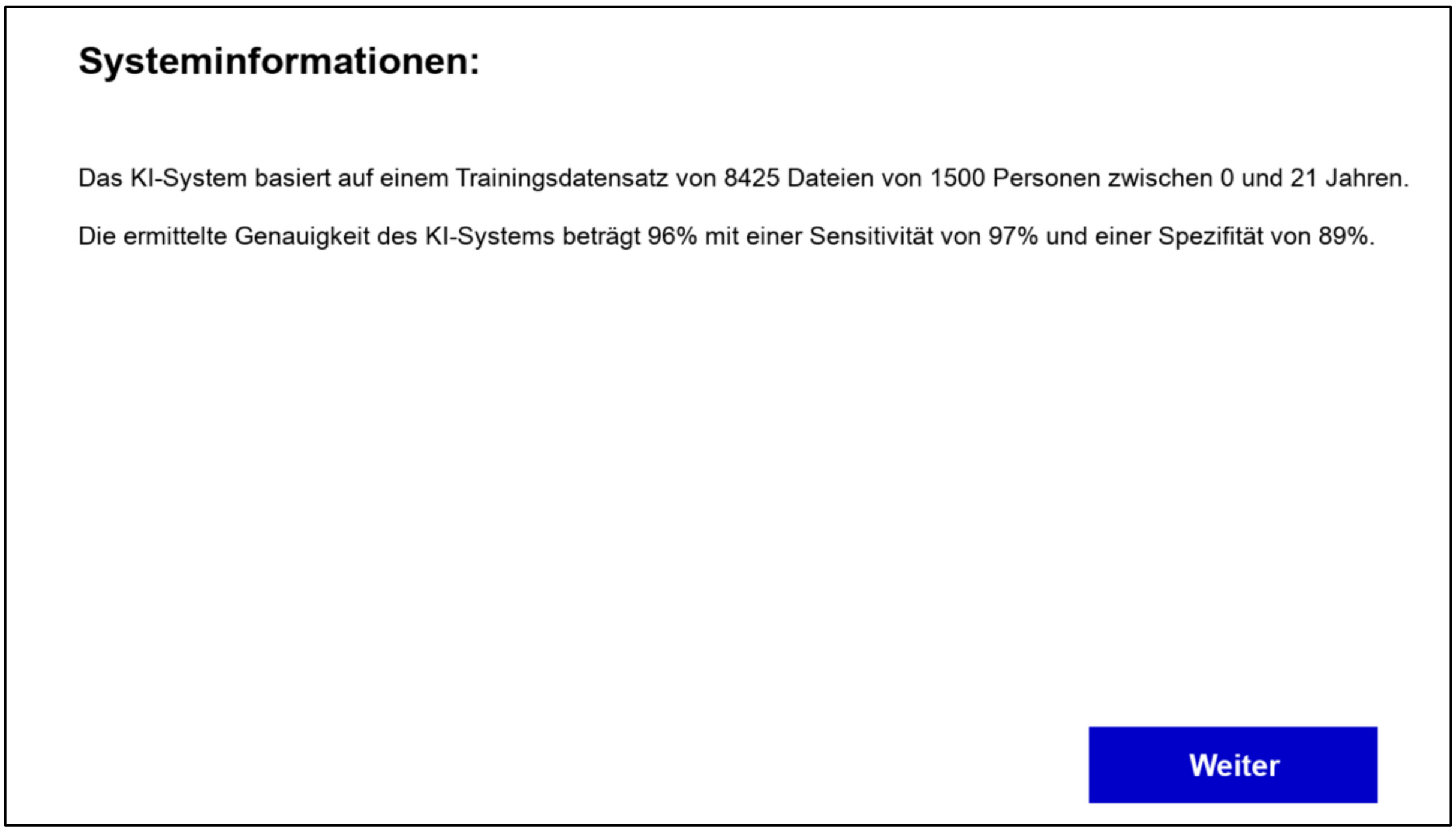

Figure S6. System information part 2. “The AI system is based on a training dataset consisting of 8,425 files from 1,500 individuals between the ages of 0 and 21. The determined accuracy of the AI system is 96%, with a sensitivity of 97% and a specificity of 89%.”

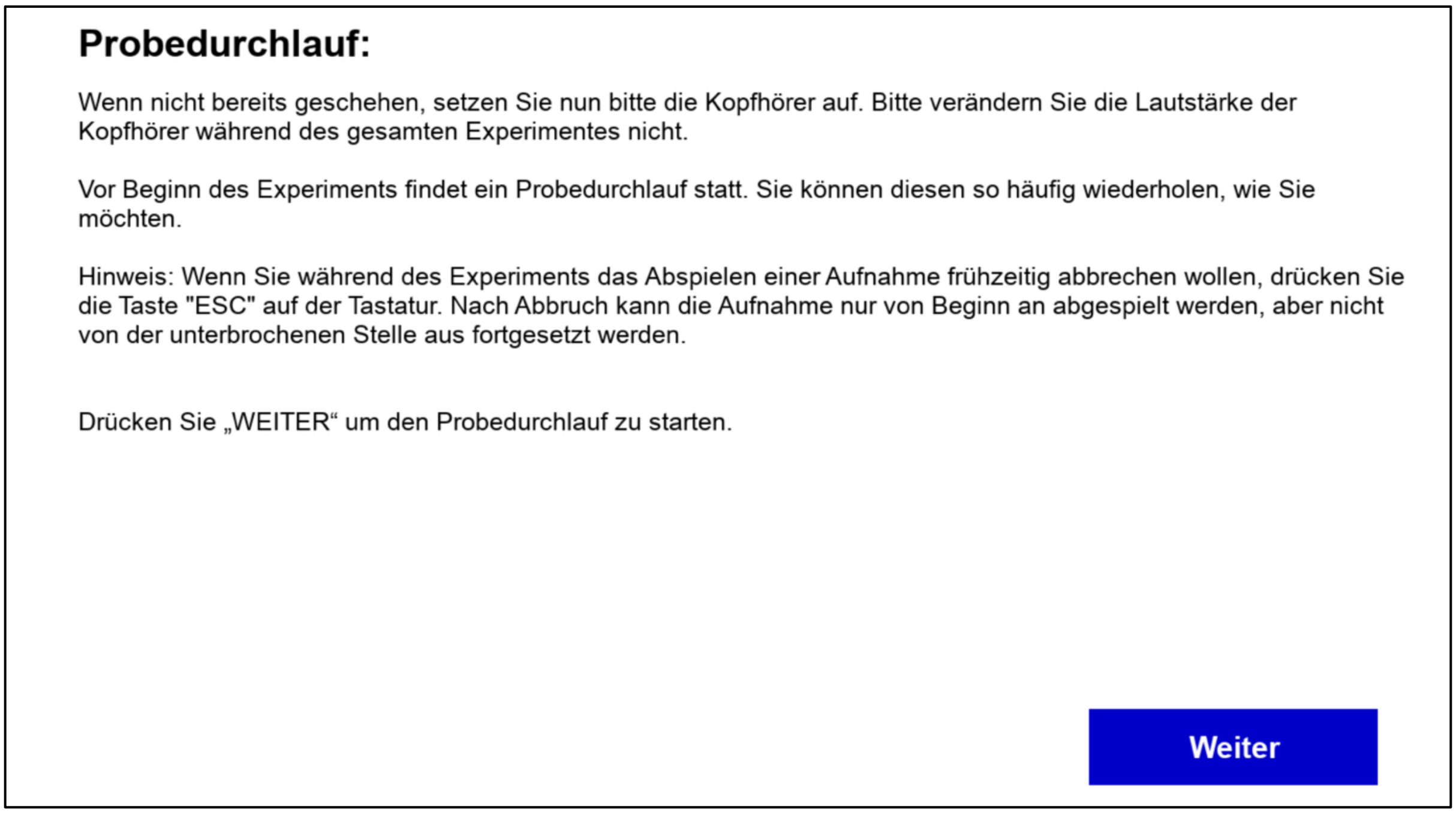

Figure S7. Practice trial. “If you haven't already, please put on your headphones now. Do not change the volume of the headphones during the entire experiment. Before the experiment begins, a practice trial will take place. You may repeat this as many times as you wish. Note: If you want to stop the playback of a recording early during the experiment, press the ‘ESC’

key on your keyboard. After cancellation, the recording can only be played again from the beginning, not from the point where it was interrupted. Press ‘CONTINUE’ to start the practice trial.”

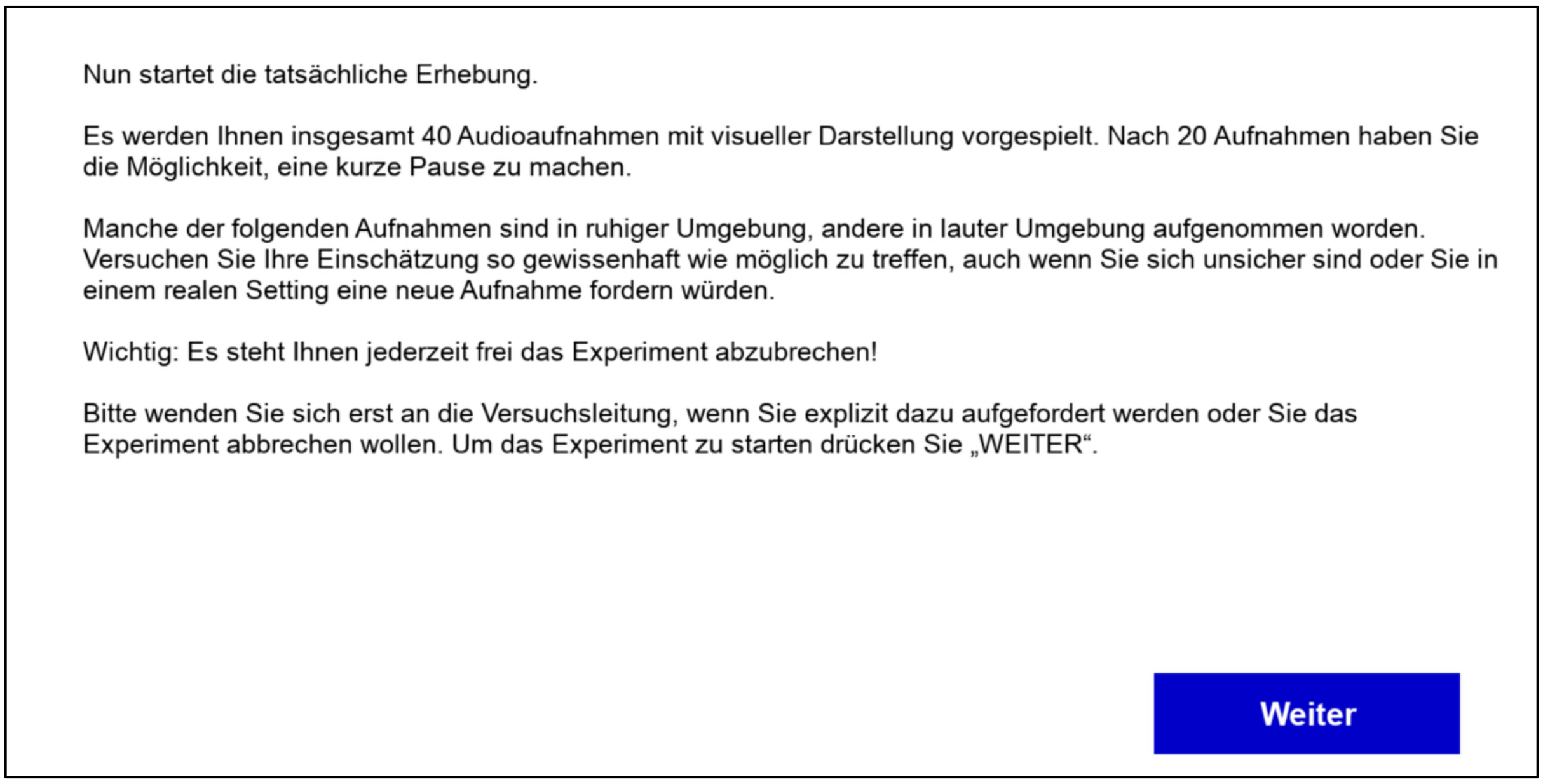

Figure S8. Instruction before start of experiment. “You will be presented with a total of 40 audio recordings with visual representation. After 20 recordings, you will have the opportunity to take a short break. Some of the upcoming recordings were made in quiet environments, others in noisy ones. Please try to make your assessments as conscientiously as possible, even if you feel uncertain or would request a new recording in a real-life setting. Important: You are free to stop the experiment at any time! Please only contact the experimentor if you are explicitly instructed to do so or if you wish to withdraw from the experiment. To start the experiment, press ‘CONTINUE.’”

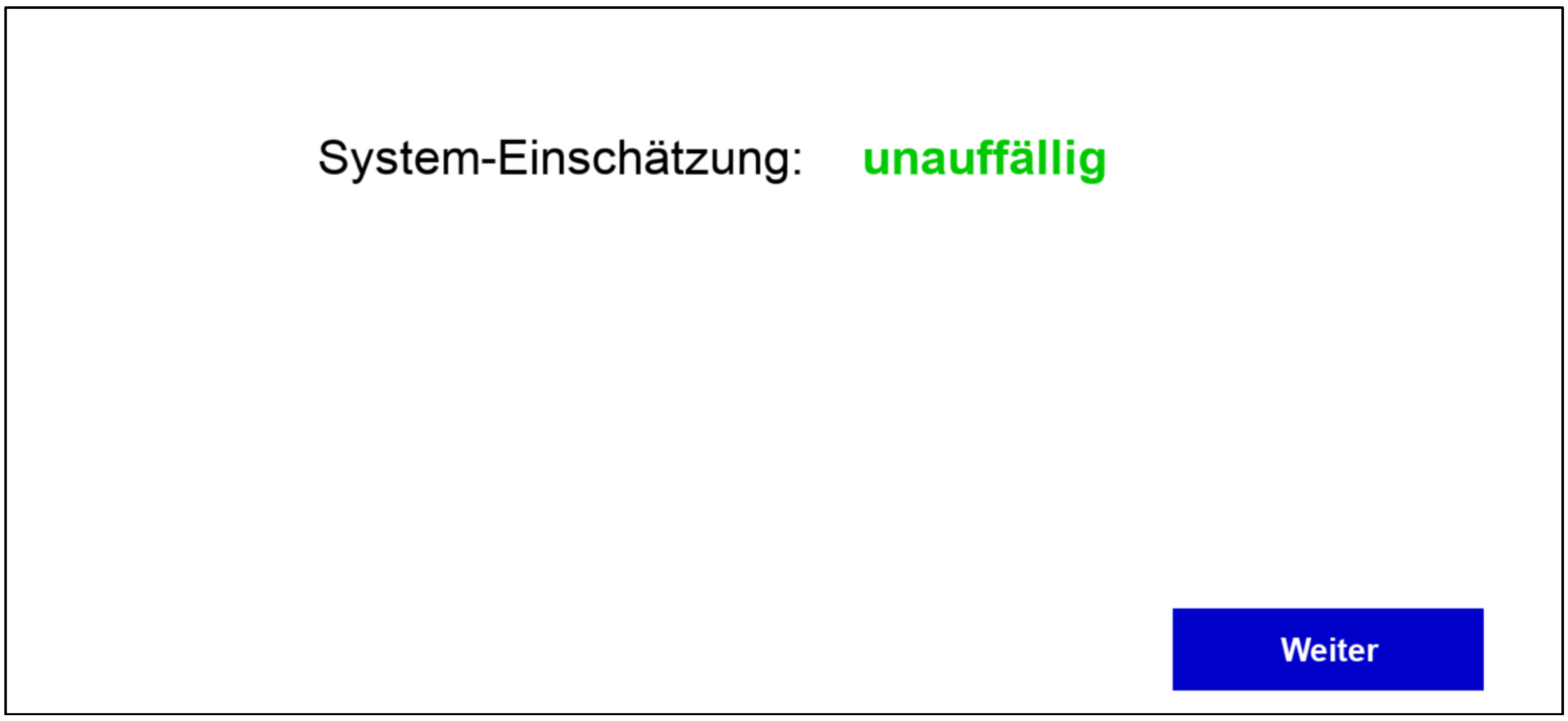

Figure S9. Sample system recommendation. “System-recommendation: normal.” Note: “Abnormal” was written in red.

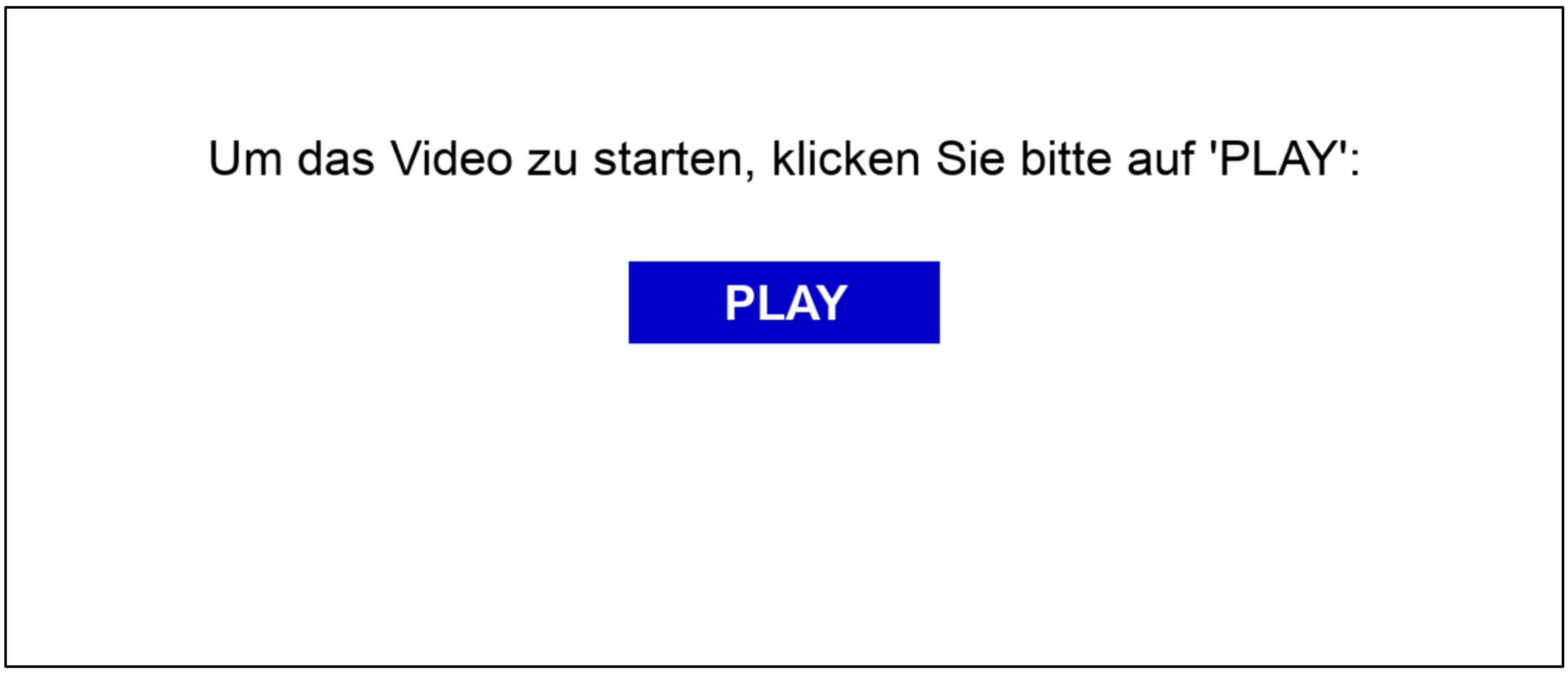

Figure S10. Play auscultation file. Click “PLAY” to start the video.

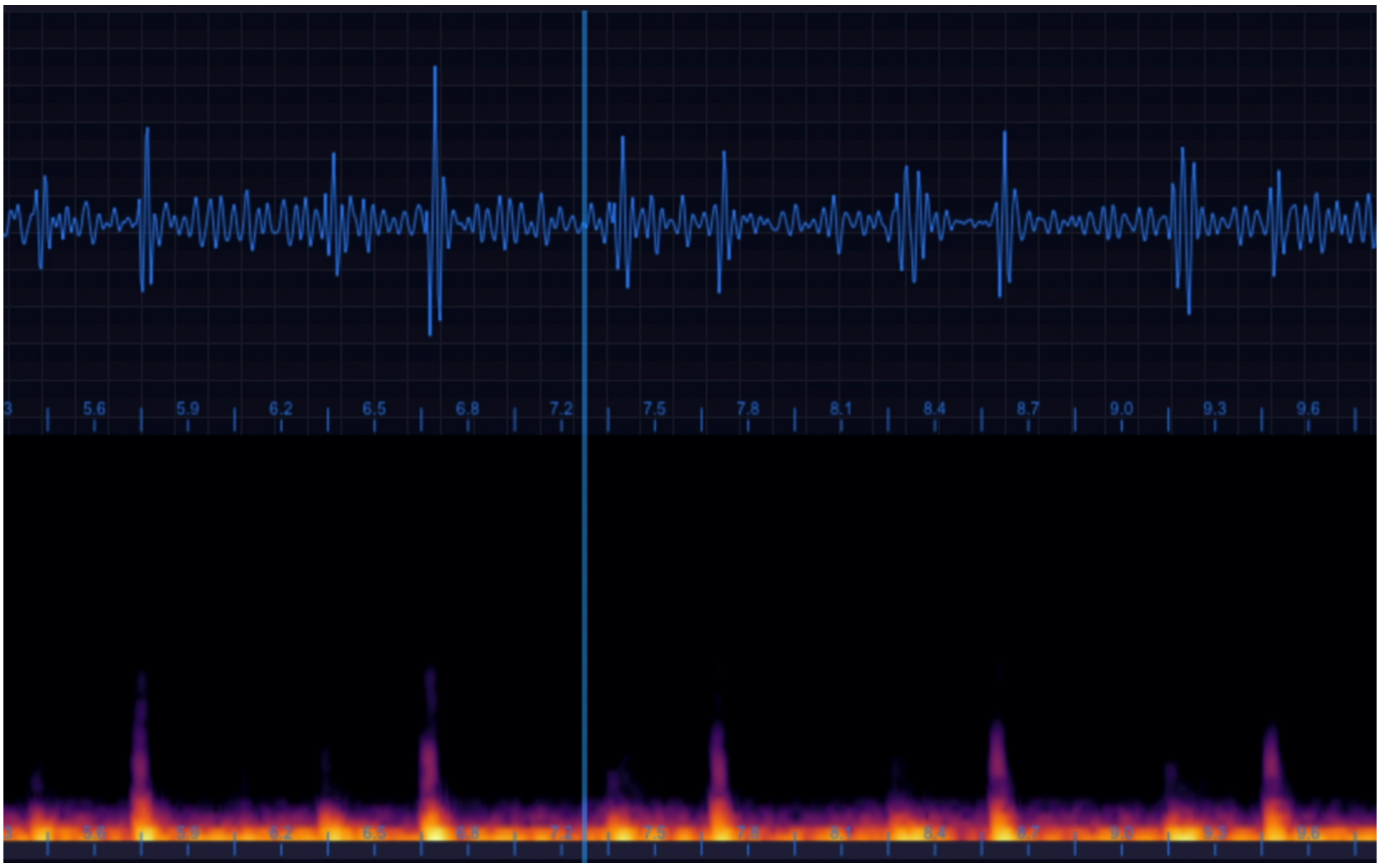

Figure S11. Sample visualization of the heart sounds.

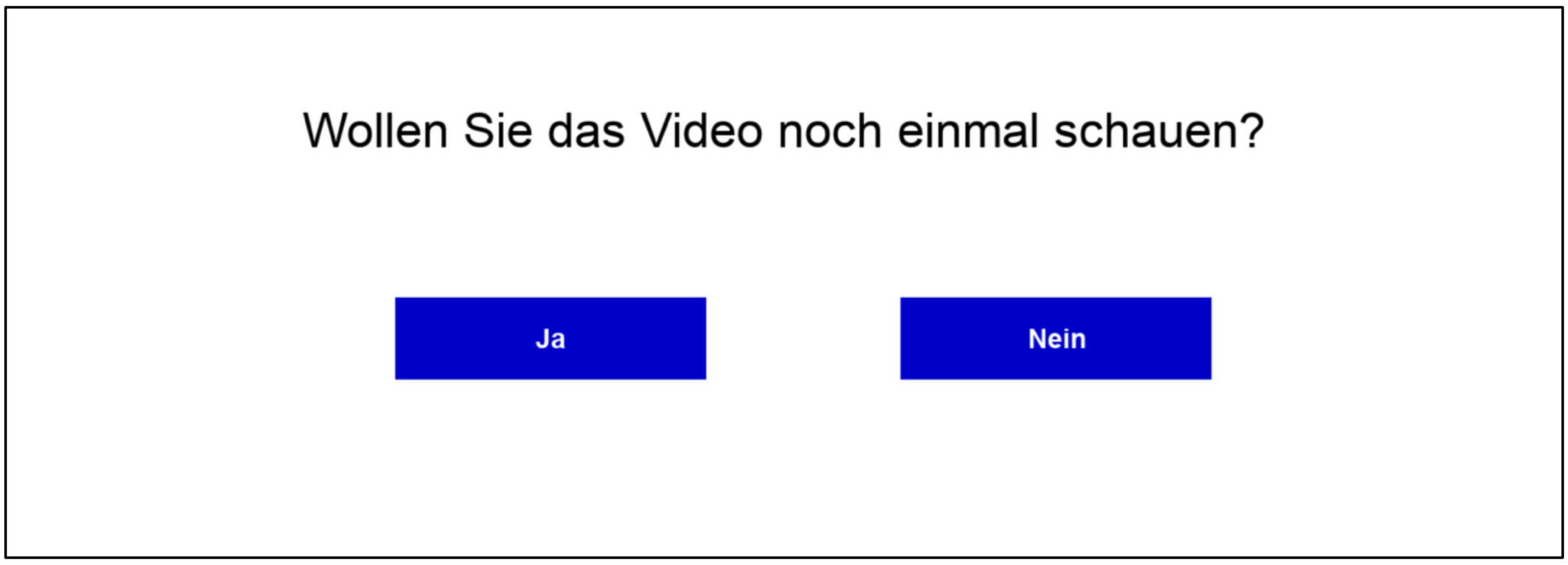

Figure S12. Replay option. After each video, participants were presented with the question: “Do you want to replay the video?” with response options “Yes” or “No”. If participants selected “Yes”, the video was replayed in full, after which the same question was posed again. This process repeated until the participant selected “No”, at which point the trial proceeded. Participants then were asked to respond to the questions as displayed in Table S1.

Items Used in the Study

In the following we report the items used in the experiment and in the post-experimental survey. Due to space restrictions, we did not report all results of the post-experimental survey in the manuscript, but we provide a correlation table at the end of the online supplement (Table S15). Additional results and analyses can be made available upon request.

Table S1. *Original German items used in the experiment and their English translation. Response options are displayed in italics. Note, perceived usefulness is not reported in the main manuscript.*

**Concepts**	**German items and *response options***	**English Translation of items and *response options***
Classification	Klassifizieren Sie bitte die Aufnahme:<br>○ *Unauffällig*<br>○ *Auffällig*	Please classify the recording:<br>○ *Normal*<br>○ *Abnormal*
Diagnostic confidence	Wie sicher sind Sie sich in Hinblick auf den festgelegten Befund?<br>○ *Überhaupt nicht sicher - ich rate*<br>○ *Nicht sehr sicher*<br>○ *Einigermaßen sicher*<br>○ *Ziemlich sicher*<br>○ *Absolut sicher*	How confident are you in your classification?<br>○ *Not confident at all – I am guessing*<br>○ *Not very confident*<br>○ *Somewhat confident*<br>○ *Fairly confident*<br>○ *Absolutely confident*
Trust in system support	Ich vertraue dem System.<br>○ *Stimme gar nicht zu*<br>○ *Stimme eher nicht zu*<br>○ *Neutral*<br>○ *Stimme eher zu*<br>○ *Stimme vollkommen zu*	I trust the system<br>○ *Strongly disagree*<br>○ *Disagree*<br>○ *Neutral*<br>○ *Agree*<br>○ *Strongly agree*
Perceived usefulness	Ich finde das System für diese Aufgabe sehr nützlich.<br>○ *Stimme gar nicht zu*<br>○ *Stimme eher nicht zu*<br>○ *Neutral*<br>○ *Stimme eher zu*<br>○ *Stimme vollkommen zu*	I consider the system very useful for this task.<br>○ *Strongly disagree*<br>○ *Disagree*<br>○ *Neutral*<br>○ *Agree*<br>○ *Strongly agree*

Table S2. *Original German items and English translation used in the post-experimental survey. Response options are provided in italics.*

Concept	German items and *response options*	English Translation of items and *response options*
Age	Wie alt sind Sie?<br>*[Offene Texteingabe]*	How old are you?<br>*[Open text input]*
Gender	Bitte geben Sie ihr Geschlecht an.<br>○ *Männlich*<br>○ *Weiblich*<br>○ *Divers*	Please enter your gender.<br>○ *Male*<br>○ *Female*<br>○ *Non-binary*
Specialty	Bitte geben Sie an, in welchem medizinischen Fachbereich Sie arbeiten.<br>*[Offene Texteingabe]*	Please indicate the medical specialty in which you work.<br>*[Open text input]*
Position	Bitte geben Sie Ihre Berufsbezeichnung bzw - position an.<br>• *PJler*<br>• *Assistenzärzt*in*<br>• *Fachärzt*in*<br>• *Funktionsoberärzt*in*<br>• *Oberärzt*in*<br>• *Chefärzt*in*<br>• *Niedergelassene/r Ärzt*in*<br>• *Weiterbildungsassistent*in*<br>• *Angestellte/r Ärzt*in*<br>• *Sonstige*	Please indicate your professional title or position.<br>• *Final-year medical student*<br>• *Junior physician / resident*<br>• *Specialist physician*<br>• *Specialist physician with specific functional responsibilities*<br>• *Senior physician*<br>• *Chief physician*<br>• *Physician in private practice*<br>• *Physician in training (specialty qualification)*<br>• *Employed physician*<br>• *Other*
Work experience	Wie viele Jahre Berufserfahrung haben Sie?<br>*[Offene Texteingabe]*	How many years of professional experience do you have?<br>*[Open text input]*
Auscultation ability	Wie würden Sie allgemein Ihre eigene Auskultationsfähigkeit einschätzen?<br>○ *Sehr schlecht*<br>○ *Schlecht*<br>○ *Mittelmäßig*<br>○ *Gut*<br>○ *Sehr gut*	How would you generally rate your own auscultation skills?<br>○ *Very poor*<br>○ *Poor*<br>○ *Average*<br>○ *Good*<br>○ *Very good*

Experience with digital auscultation	Haben Sie bereits Erfahrung mit digitaler Auskultation gemacht?<br>*Ja*<br>*Nein*	Do you have any experience with digital auscultation?<br>*Yes*<br>*No*
Affinity for Technology Interaction (ATI) Scale (Franke, Attig, & Wessel, 2018[1])	1. Ich beschäftige mich gern genauer mit technischen Systemen.<br>2. Ich probiere gern die Funktionen neuer technischer Systeme aus.<br>3. In erster Linie beschäftige ich mich mit technischen Systemen, weil ich muss.<br>4. Wenn ich ein neues technisches System vor mir habe, probiere ich es intensiv aus.<br>5. Ich verbringe sehr gern Zeit mit dem Kennenlernen eines neuen technischen Systems.<br>6. Es genügt mir, dass ein technisches System funktioniert, mir ist es egal, wie oder warum.<br>7. Ich versuche zu verstehen, wie ein technisches System genau funktioniert.<br>8. Es genügt mir, die Grundfunktionen eines technischen Systems zu kennen.<br>9. Ich versuche, die Möglichkeiten eines technischen Systems vollständig auszunutzen.<br>o *Stimmt gar nicht*<br>o *Stimmt weitgehend nicht*<br>o *Stimmt eher nicht*<br>o *Stimmt eher*<br>o *Stimmt weitgehend*<br>o *Stimmt völlig*	1. I like to occupy myself in greater detail with technical systems.<br>2. I like testing the functions of new technical systems.<br>3. I predominantly deal with technical systems because I have to.*<br>4. When I have a new technical system in front of me, I try it out intensively.<br>5. I enjoy spending time becoming acquainted with a new technical system.<br>6. It is enough for me that a technical system works; I don't care how or why.*<br>7. I try to understand how a technical system exactly works.<br>8. It is enough for me to know the basic functions of a technical system.*<br>9. I try to make full use of the capabilities of a technical system.<br>o *Completely disagree*<br>o *Largely disagree*<br>o *Slightly disagree*<br>o *Slightly agree*<br>o *Largely agree*<br>o *Completely agree*
Qualitative Assessment	1. Ist Ihnen bei der Bearbeitung des Experiments etwas aufgefallen?<br>2. Hatten Sie das Gefühl, dass sich das System verändert hat? Wenn ja, zu welchem Zeitpunkt kam dieses Gefühl auf?<br>*[Offene Texteingabe]*	1. Did you notice anything during the experiment?<br>2. Did you have the impression that the system changed? If so, at what point did you become aware of this?<br>*[Open text input]*

Table S3. *Additional items used in the post-experimental survey, which are not reported in the manuscript. Items and response options are provided in German as used in the study and in an English Translations. Asterisks indicate inverted items. Response options are displayed in italics. Results can be made available upon request.*

**Concept**	**German items and *response options***	**English Translation of items and *response options***
Trust in Automation (selected items from Körber, 2019[2])	1. Das System ist imstande Situationen richtig einzuschätzen.<br>2. Das System arbeitet zuverlässig.<br>3. Ein Ausfall des Systems ist wahrscheinlich.<br>4. Das System kann wirklich komplizierte Aufgaben übernehmen.<br>5. Das System könnte stellenweise einen Fehler machen.<br>6. Ich bin überzeugt von den Fähigkeiten des Systems.<br>7. Mir war durchgehend klar, in welchem Zustand sich das System befindet.<br>8. Ich konnte nachvollziehen, warum etwas passiert ist.<br>9. Zu erkennen, was das System als Nächstes macht, ist schwer.<br>10. Automatisierte Systeme funktionieren generell gut.<br>11. Ich kenne bereits ähnliche Systeme.<br>12. Ich habe ähnliche Systeme bereits genutzt.<br>13. Bei unbekannten automatisierten Systemen sollte man eher vorsichtig sein.<br>14. Das System reagiert unvorhersehbar.<br>15. Ich vertraue einem System eher, als dass ich ihm misstraue.<br>16. Ich vertraue dem System.<br>17. Ich kann mich auf das System verlassen.<br><br>o *Stimme gar nicht zu*<br>o *Stimme eher nicht zu*<br>o *Weder noch*<br>o *Stimme eher zu*<br>o *Stimme vollkommen zu*	1. The system is capable of interpreting situations correctly.<br>2. The system works reliably.<br>3. A system malfunction is likely.*<br>4. The system is capable of taking over complicated tasks.<br>5. The system might make sporadic errors.*<br>6. I am confident about the system's capabilities.<br>7. The system state was always clear to me.<br>8. I was able to understand why things happened.<br>9. It's difficult to identify what the system will do next.*<br>10. Automated systems generally work well.<br>11. I already know similar systems.<br>12. I have already used similar systems.<br>13. One should be careful with unfamiliar automated systems.*<br>14. The system reacts unpredictably.*<br>15. I rather trust a system than I mistrust it.<br>16. I trust the system.<br>17. I can rely on the system.<br><br>o *Strongly disagree*<br>o *Rather disagree*<br>o *Neither disagree nor agree*<br>o *Rather agree*<br>o *Strongly agree*
System Usefulness (one	<u>Lokale Nützlichkeit (self developed):</u>	<u>Local Usefulness(self developed):</u>

self developed item + eight selected items from Venkatesh & Davis, 2000[3])	1. Das System war nützlich für die Auskultation im Experiment.<br><u>Nutzungsintention:</u><br>1. Unter der Annahme, dass ich Zugang zu dem System habe, beabsichtige ich, es zu nutzen.<br>2. Wenn ich Zugang zu dem System habe, werde ich es auch nutzen.<br><br><u>Wahrgenommene Nützlichkeit:</u><br>1. Die Nutzung des Systems würde meine Leistung auf der Arbeit verbessern.<br>2. Der Einsatz des Systems bei meiner Arbeit würde meine Produktivität erhöhen.<br>3. Die Nutzung des Systems würde meine Effektivität bei der Arbeit erhöhen.<br>4. Ich empfinde das System als nützlich für meine Arbeit.<br><br><u>Job Relevanz:</u><br>1. Für meine Arbeit wäre die Nutzung des Systems wichtig.<br>2. Für meine Arbeit wäre die Nutzung des Systems relevant.<br>o *Stimme überhaupt nicht zu*<br>o *Stimme nur wenig zu*<br>o *Stimme eher nicht zu*<br>o *Neutral*<br>o *Stimme eher zu*<br>o *Stimme vorwiegend zu*<br>o *Stimme voll und ganz zu*	1. The system was useful for auscultation in the experiment.<br><u>Intention to use:</u><br>1. Assuming I have access to the system, I intend to use it.<br>2. Given that I have access to the system, I predict that I would use it.<br><br><u>Perceived Usefulness:</u><br>1. Using the system improves my performance in my job.<br>2. Using the system in my job increases my productivity.<br>3. Using the system enhances my effectiveness in my job.<br>4. I find the system to be useful in my job.<br><br><u>Job relevance:</u><br>1. In my job, usage of the system is important.<br>2. In my job, usage of the system is relevant.<br>o *Strongly disagree*<br>o *Moderately disagree*<br>o *Somewhat disagree*<br>o *Neutral*<br>o *Somewhat agree*<br>o *Moderately agree*<br>o *Strongly agree*

Stimulus Selection

The following section outlines the procedure used to select the 40 audio recordings from the CirCor dataset[4] employed in the current study. The CirCor dataset comprises 3,163 recordings collected from 942 individuals. For each individual, up to four recordings were captured at standardized auscultation sites (aortic, pulmonary, mitral, and tricuspid valves). In addition to the audio data, the dataset contains demographic information (e.g., age, sex), details regarding the recording context (clinical vs. non-clinical), and annotations by medical experts. These annotations include the presence or absence of heart murmurs (classified as present, absent, or unknown) and an independent clinical outcome rating (normal or abnormal), based on cardiological assessment.

For the purposes of this study, the dataset was first screened for completeness. Only cases with a clearly defined murmur classification—either "present" or "absent"—were included. Cases labeled as "unknown" ($n = 68$) were excluded, resulting in 874 eligible cases. Subsequently, we cross-referenced the murmur classification with the independently determined clinical outcome. The murmur classification, assigned by trained professionals using digital auscultation (DA), differentiated between cases in which at least one pathological sound was detected (murmur present) and those with no pathological sounds identified (murmur absent). The clinical outcome, on the other hand, reflected a comprehensive assessment conducted by pediatric cardiologists, who did not have access to the digital auscultation recordings or any computer-assisted analysis. Their assessment was based on medical history, physical examination, analog auscultation, and echocardiographic results. Discrepancies may thus arise between murmur presence in DA and clinical outcome, particularly in cases where subtle murmurs were not detected via traditional auscultation. The cardiologists involved in determining the outcome were not involved in recording or annotating the digital auscultation data.

For this study, only cases with full agreement between the murmur classification and clinical outcome were retained, representing the gold standard for ground truth labeling. Cases where a murmur was absent but the clinical outcome was abnormal ($n = 263$) and those where a murmur was present but the outcome was normal ($n = 29$) were excluded. The resulting filtered dataset included 582 consistent cases.

Next, these cases were categorized by age group according to the classification system used in the dataset distinguishing between newborns (0–27 days), infants/toddlers (28 days to 1 year), children (1–11 years), adolescents (12–18 years), and young adults (19–21 years). Cases lacking age data (*n* = 4) and pregnant patients (*n* = 54) were excluded, yielding a final dataset of 524 cases.

For pilot testing purposes, 52 recordings were selected to ensure a balanced representation of normal and abnormal cases across age groups. Twelve of these recordings (with the intention to include three of them in each of the four blocks) were reused from a prior study[1] with a similar design, where audio files were already piloted, and difficulty was already assessed. These existing recordings had been augmented with background noise elements typical of clinical settings (e.g., conversations, monitor sounds) to increase diagnostic difficulty. The remaining 40 recordings were randomly sampled from the filtered dataset using a random number generator (https://www.random.org/). The selected sample comprised 24 adolescents, 23 children, 4 toddlers, and 1 newborn. For individuals with multiple recordings, only those lasting at least 10 seconds were considered. In abnormal cases, the acoustically most prominent recording ("best heard at") was selected; in normal cases, the clearest audio file was chosen (based on the the independent rating of two researchers).

All selected recordings were uploaded into an AI-tool that visualized the soundfiles as phonocardiograms (see Figure S11), captured via screencast, and synchronized in iMovie with their original audio to increase sound quality. This created standardized audiovisual stimuli suitable for presentation in the experimental paradigm.

The pilot study was conducted in two phases to calibrate stimulus difficulty and finalize the block structure. The average age of the four pilot participants—all female medical students—was *M* = 23.5 years (*SD* = 0.58). In the first phase, two fifth-year medical students evaluated all 52 recordings without AI assistance to assess initial diagnostic difficulty. Each recording was labeled as either normal or abnormal, and rated for difficulty on a 5-point rating scale from 1 = "Very easy", over 2 = "Rather easy", 3 = "Moderate", 4 = "Rather difficult", to 5 = "Very difficult". Based on these ratings, the recordings were preliminarily sorted to ensure

---

[1] Note, participants of this prior study have been excluded in the present data collection.

that each of the four experimental blocks (T1–T4) would contain an equal number of recordings and reflect a comparable difficulty level.

In the second phase, two additional fifth-year medical students evaluated this pre-sorted set using the same procedure. These secondary ratings were used to refine block composition and achieve further balance in difficulty across blocks. Recordings that were consistently rated as very easy ($M < 1.5$, $n = 4$) or very difficult ($M > 4.5$, $n = 4$) across both pilot groups were removed to avoid floor and ceiling effects.

From the remaining 43 recordings, a final set of 40 stimuli was selected and evenly distributed across the four stimulus blocks, with three of the preprocessed stimuli in each blok. The composition of blocks was fixed (each block contained always the same 10 recordings), but the presentation order was randomized during the experiment to control for sequence effects. Each block had a mean difficulty level of approximately 3 on the 5-point Likert scale and an average diagnostic accuracy of 73%, mirroring expert performance levels in auscultation tasks[5]. Specifically, the difficulty ratings were as follows: Block T1 (M = 3.03, SD = 1.37), Block T2 (M = 3.03, SD = 1.17), Block T3 (M = 3.13, SD = 1.09), and Block T4 (M = 3.05, SD = 1.11). Performance levels across blocks were similarly stable: T1 (M = 0.70, SD = 0.46), T2 (M = 7.25, SD = 0.45), T3 (M = 7.25, SD = 0.45), and T4 (M = 7.0, SD = 0.46). Within each block, 50% of recordings were abnormal (containing a murmur), and 50% were normal. The final sample used in the experiment included 17 adolescents (eight normal, nine abnormal), 19 children (nine normal, ten abnormal), three toddlers (two normal, one abnormal), and one newborn (normal).

To simulate progressive system reliability degradation, the four stimulus blocks differed in their programmed AI error rates. Each block varied in the number and type of diagnostic errors assigned to the AI system. Error distributions were determined using an online random number generator (https://www.random.org/). Accordingly, errors were assigned at the level of individual audio files rather than being determined by their position within the stimulus block. In Block 1, the AI produced four true negatives, five true positives, and one false positive. Block 2 included two true negatives, four true positives, three false positives, and one false negative. Block 3 presented three true negatives, two true positives, three false positives, and two false negatives. Block 4 contained only false classifications—five false positives and five false negatives. Additionally, in the final 10 trials (Block 4), a deliberate asynchrony was introduced. Each audio file was sampled in iMovie with a non-corresponding

phonocardiogram video taken from a different recording from the original data set[6]. While their start and end points were synchronized, the audio and visual content was mismatched, simulating a system malfunction. The final set of stimuli is available at https://github.com/IKI-Lab/human_oversight_auscultation.

Supplementary Data Analysis

This supplement presents additional sensitivity analysis that test the robustness of the findings reported in the manuscript as well as extended explorative analysis to provide readers with a more comprehensive understanding of the data. Analysis code is available here: https://osf.io/836ny/?view_only=3a1631a54edd489197a8b3b9e4322fa0. Main analysis was conducted in RStudio (version 4.3.2) using the following packages: tidyverse[7], descr[8], sjmisc[9], sjlabelled[10], sjstats[11], sjPlot[12], easystats[13], ggplot2[14], GGally[15], car[16], psych[17],ggpubr[18], emmeans[19], apaTables[20], rstudioapi[21], effectsize[22], dplyr[23], weights[24], lme4[25], lmerTest[26], lsr[27], rstatix[28], readxl[29], gtsummary[30], afex[31]. In the following, we first provide the additional sensitivity analysis to validate the results reported in the manuscript. Then we provide exploratory analysis and additional demographic information of the study sample and the subgroup distribution. At the end, for completeness and further data exploration, we provide a correlational table including descriptive statistics (mean, standard deviation) and intercorrelations of variables assessed in this experiment (see Table S15).

Sensitivity Analysis

Here, we report the sensitivity analysis for the main findings in the manuscript, i.e. performance (after and before AI recommendation), confidence (after and before AI recommendation), and trust in AI. To account for potential nested influences, we additionally tested interaction effects using hierarchical linear models with participant ID as a random factor. All models were fitted with treatment coding, with Group 1 and Block 1 as reference categories. The main findings remained robust in the sensitivity analysis, even when we additionally controlled for participants' experience and self-assessed auscultation skill. Results from extended models (that separately included experience and self-assessed auscultation skill) are reported only when experience or self-assessed auscultation skill showed a significant effect. There was no change in the interpretation of our results, when experience and auscultation skill were included together in one model. For the binary

performance outcome, re-transformed estimates are presented as odds ratios with corresponding 95% confidence intervals (CI). The significance threshold was set at $\alpha = .05$, and two-tailed p-values are reported (i.e., non-halved *p*-values).

Performance

Performance after AI recommendation

A generalized linear mixed model with a binomial distribution and logit link was used to investigate participants' performance accuracy (Correct Final). As fixed effects we applied group (AI-First vs. AI-Second), system reliability in the four blocks (90%, 60%, 50%, 0%), and their interaction, with a random intercept for participant-ID to account for within-subject variability.

Results are consistent with our main analysis (see Table S4). There was a significant main effect of system reliability, indicating a decrease in performance over the course of the four blocks. Specifically, performance was significantly lower in block 2 (60%), block 3 (50%), and block 4 (0%), compared to the reference level in block 1 (90%).

A significant interaction emerged between group and system reliability in block 4 (0%), indicating that performance here was significantly lower in the AI-Second group relative to the AI-First group.

Experience (p = .862) and self-assessed auscultation skill (p = .111) did not reach significance when included into the model.

Table S4. *Logistic mixed-effects model for the chance of correct final classifications. The model includes fixed effects for experimental group, block, and their interaction, with a random intercept for participant ID (prob_number). Odds ratios, 95% confidence intervals (CI), and p-values are reported. Significant effects are highlighted in bold font.*

Logistic Mixed-Effects Model: Correct Final

	**Correct Final (1 = correct)**		
Predictors	*Odds Ratios*	*CI*	*p*
(Intercept)	4.30	3.06 – 6.02	**<0.001**
Group [AI-Second]	1.09	0.67 – 1.77	0.731

Block T2 [60%]	0.61	0.40 – 0.94	**0.024**
Block T3 [50%]	0.40	0.26 – 0.60	**<0.001**
Block T4 [0%]	0.43	0.28 – 0.65	**<0.001**
Group [AI-Second] × Block [T2]	1.07	0.58 – 1.98	0.829
Group [AI-Second] × Block [T3]	1.13	0.62 – 2.05	0.692
Group [AI-Second] × Block [T4]	0.54	0.30 – 0.98	**0.044**
**Random Effects**			
σ^2	3.29		
$\tau_{00\ prob\_number}$	0.06		
ICC	0.02		
N_{prob_number}	48		
Observations	1920		
Marginal R^2 / Conditional R^2	0.059 / 0.076		

Performance before AI recommendation

A generalized linear mixed model (GLMM) with a binomial distribution and logit link was conducted to examine the likelihood of a correct final decision before participants within the *AI-Second* group received the AI recommendation in the four blocks (90%, 60%, 50%, 0%). The model included a random intercept for participant-ID to account for repeated measurements.

Results are consistent with our main analysis, and hence revealed no significant effects of the blocks on accuracy (see Table S5). While experience showed a statistically significant negative effect (p = .046), its inclusion did not alter the pattern or interpretation of the main results (see Table S6). In particular, the effects of the experimental blocks remained non-significant. This suggests that the observed accuracy patterns are not attributable to participants' experience levels. Self-assessed auscultation skill was not significant when included into the model instead of experience (p = .571).

Table S5. *Logistic mixed-effects model for the chance of correct final classifications before AI recommendation in the AI-second group. The model includes fixed effects for experimental block and a random intercept for participant-ID (prob_number). Odds ratios, 95% confidence intervals (CI), and p values are reported. Significant effects are highlighted in bold font.*

Logistic Mixed-Effects Model: Accuracy before AI recommendation

	**Correct Final (1 = correct)**		
Predictors	*Odds Ratios*	*CI*	*p*
(Intercept)	2.71	2.02 – 3.65	**<0.001**
Block T2 [60%]	1.19	0.79 – 1.80	0.400
Block T3 [50%]	1.25	0.83 – 1.90	0.291
Block T4 [0%]	1.35	0.88 – 2.05	0.166
**Random Effects**			
σ^2	3.29		
$\tau_{00\ prob\_number}$	0.04		
ICC	0.01		
N_{prob_number}	24		
Observations	960		
Marginal R^2 / Conditional R^2	0.004 / 0.014		

Table S6. *Logistic mixed-effects model for the chance of correct final classifications before AI recommendation in the AI-second group, controlling for participants work experience. The model includes fixed effects for experimental block and a random intercept for participant-ID (prob_number). Odds ratios, 95% confidence intervals (CI), and p values are reported. Significant effects are highlighted in bold font*

Logistic Mixed-Effects Model : Accuracy Before AI Recommendation + Experience

	**Correct Final (1 = correct)**		
Predictors	*Odds Ratios*	*CI*	*p*

(Intercept)	2.95	2.18 – 3.99	**<0.001**
Block T2 [60%]	1.19	0.79 – 1.80	0.400
Block T3 [50%]	1.25	0.83 – 1.90	0.291
Block T4 [0%]	1.35	0.88 – 2.05	0.166
Experience	0.99	0.98 – 1.00	**0.046**
**Random Effects**			
σ^2	3.29		
$\tau_{00\ prob\_number}$	0.01		
ICC	0.00		
N_{prob_number}	24		
Observations	960		
Marginal R^2 / Conditional R^2	0.010 / 0.013		

Confidence

Confidence after AI recommendation

A linear mixed-effects model was conducted to examine the effects of decreasing system-reliability in the four blocks (90%, 60%, 50%, 0%) on participants' *confidence ratings* after receiving the system recommendation. The model included block as fixed effect and participant ID as random effect to account for individual differences.

Results are consistent with our main analysis (see Table S7). The results indicate a decline in confidence ratings with decreasing system reliability, particularly in blocks three (50% system reliability) and four (0% system reliability).

Experience ($p = .223$) and self-assessed auscultation skill ($p = .127$) did not reach significance when included into the model.

Table S7. *Linear mixed-effects model for the participants' confidence ratings after receiving the AI recommendation. The model includes fixed effects for experimental block and a*

random intercept for participant ID (prob_number). Regression estimates, 95% confidence intervals (CI), and p values are reported. Significant effects are highlighted in bold font.

Linear Mixed Model: Confidence Ratings

	**Confidence (after AI recommendation)**		
Predictors	*Estimates*	*CI*	*p*
(Intercept)	3.39	3.26 – 3.52	**<0.001**
Block T2 [60%]	-0.04	-0.17 – 0.09	0.525
Block T3 [50%]	-0.29	-0.42 – -0.16	**<0.001**
Block T4 [0%]	-0.36	-0.48 – -0.23	**<0.001**
**Random Effects**			
σ^2	1.03		
$\tau_{00\ prob\_number}$	0.11		
ICC	0.10		
N_{prob_number}	48		
Observations	1920		
Marginal R^2 / Conditional R^2	0.020 / 0.114		

Confidence before AI recommendation

To investigate baseline differences of participants in the AI-second group, we fitted a linear mixed-effects model to estimate participants' confidence ratings before the AI recommendation based four blocks (90%, 60%, 50%, 0%). The model included random intercepts for participant ID to account for individual differences.

Results support our conclusions from the main anaylsis (see Table S8). There was a significant main effect of block, indicating that confidence levels varied across experimental blocks. Specifically, compared to block 1 (90%) confidence dropped significantly in block 3.

Experience (p = .660) and self-assessed auscultation skill (p = .062) did not reach significance when included into the model.

Table S8. *Linear mixed-effects model for participants' confidence ratings before receiving the AI recommendation (in the AI-second group). The model includes fixed effects for experimental block and a random intercept for participant ID (prob_number). Regression estimates, 95% confidence intervals (CI), and p values are reported. Significant effects are highlighted in bold font.*

Linear Mixed Model: Confidence Ratings (AI-Second Group)

	**Confidence (before AI recommendation)**		
Predictors	*Estimates*	*CI*	*p*
(Intercept)	3.22	3.01 – 3.43	**<0.001**
Block T2 [60%]	0.12	-0.06 – 0.29	0.194
Block T3 [50%]	-0.31	-0.48 – -0.13	**0.001**
Block T4 [0%]	-0.15	-0.32 – 0.03	0.104
**Random Effects**			
σ^2	0.96		
$\tau_{00\ prob\_number}$	0.19		
ICC	0.16		
N_{prob_number}	24		
Observations	960		
Marginal R^2 / Conditional R^2	0.022 / 0.181		

Trust

A linear mixed-effects model was conducted to examine the effects of group (AI-First vs. AI-Second), and system reliability in the four blocks (90%, 60%, 50%, 0%), and their interaction on trust ratings during the experiment. To account for individual effects we modelled participant ID as random effect.

Results are consistent with our main analysis (see Table S9). Overall, participants' trust decreased over time, with a significant drop observed in block 4, regardless of group assignment. There was no significant group difference nor a significant interaction, indicating that the pattern of change in trust over blocks was comparable for both the AI-first and AI-second groups.

Experience did not reach significance when included into the model (p = .058). While self-assessed auscultation skill showed a statistically significant negative effect (p = .008), its inclusion did not alter the pattern or interpretation of the main results (see Table S10). In particular, the effects of the experimental blocks remained non-significant. This suggests that the observed accuracy patterns are not attributable to participants' experience levels and auscultation skill.

Table S9. *Linear mixed-effects model for participants' trust ratings in the AI system. The model includes fixed effects for group, block, and their interaction, as well as a random intercept for participant ID (prob_number). Regression estimates, 95% confidence intervals (CI), and p values are reported. Significant effects are highlighted in bold font.*

Linear Mixed Model: Trust Ratings

	**Trust**		
Predictors	*Estimates*	*CI*	*p*
(Intercept)	3.21	2.89 – 3.53	**<0.001**
Group [AI-Second]	0.38	-0.08 – 0.83	0.106
Block T2 [60%]	0.13	-0.14 – 0.39	0.347
Block T3 [50%]	0.06	-0.20 – 0.32	0.638
Block T4 [0%]	-0.38	-0.64 – -0.11	**0.005**
Group [AI-Second] × Block [T2]	-0.13	-0.49 – 0.24	0.506
Group [AI-Second] × Block [T3]	0.19	-0.18 – 0.56	0.319
Group [AI-Second] × Block [T4]	0.02	-0.35 – 0.39	0.912
**Random Effects**			
σ^2	0.42		
τ_{00} prob_number	0.43		
ICC	0.50		
N prob_number	48		
Observations	384		
Marginal R^2 / Conditional R^2	0.087 / 0.547		

Table S10. *Linear mixed-effects model for participants' trust ratings in the AI system controlling for participants self-assessed auscultation skill. The model includes fixed effects for group, block, and their interaction, as well as a random intercept for participant ID (prob_number). Regression estimates, 95% confidence intervals (CI), and p values are reported. Significant effects are highlighted in bold font.*

Linear Mixed Model: Trust Ratings + Auscultation Skill

	**Trust**		
Predictors	*Estimates*	*CI*	*p*
(Intercept)	4.21	3.41 – 5.01	**<0.001**
Group [AI-Second]	0.35	-0.09 – 0.78	0.117
Block [T2]	0.13	-0.14 – 0.39	0.347
Block [T3]	0.06	-0.20 – 0.32	0.638
Block [T4]	-0.37	-0.64 – -0.11	**0.005**
auscultation skill	-0.33	-0.57 – -0.09	**0.008**
Group [AI-Second] × Block [T2]	-0.13	-0.49 – 0.24	0.506
Group [AI-Second] × Block [T3]	0.19	-0.18 – 0.56	0.319
Group [AI-Second] × Block [T4]	0.02	-0.35 – 0.39	0.912
**Random Effects**			
σ^2	0.42		
$\tau_{00\ prob\_number}$	0.37		
ICC	0.47		
N_{prob_number}	48		
Observations	384		
Marginal R^2 / Conditional R^2	0.154 / 0.550		

Exploratory Analysis

Inspired by the results of Ryan et al.[32] we explored the behavioral pattern of participants, and whether it was dependent on the AI's recommendation ("normal" vs. "abnormal"). Specifically, we explored whether participants were more likely to either change their opinion (AI-Second group) or override AI recommendations (AI-Second and AI-First group) when the AI classified heart sounds as "abnormal" than when they were classified as "normal". Our results show that participants in the AI-Second group were not significantly more likely to change their opinion when the AI prediction was "normal" (18%) versus "abnormal" (21%, see Table S11). Additionally, they were not significantly less likely to override "abnormal" AI predictions (see Table S12). Participants in the AI-First group were significantly more likely to override "abnormal" AI classifications than "normal" classifications (see Table S13).

Table S11. *Switching Behavior in the AI-Second Group. Switching Behavior was calculated as the change between participants opinion before and after seeing AI advice. AI recommendation reflects the advice participants received from the AI (i.e. "normal"= 0, and "abnormal"= 1).*

Logistic Model Predicting Switching Behavior in AI-Second Group

	**Switching_behavior (1 = switch)**		
Predictors	*Odds Ratios*	*CI*	*p*
(Intercept)	0.50	0.25 – 1.00	0.051
AI recommendation	1.43	0.91 – 2.25	0.117
**Random Effects**			
σ^2	3.29		
$\tau_{00\ prob\_number}$	2.19		
ICC	0.40		
N_{prob_number}	24		
Observations	453		
Marginal R^2 / Conditional R^2	0.006 / 0.403		

Table S12. *Overriding Behavior in the AI-Second Group. Overriding Behavior was calculated as the comparison between the AI prediction and the final classification. AI recommendation reflects the advice from the AI (i.e. "normal"= 0, and "abnormal"= 1).*

Logistic Model Predicting Overriding Behavior in AI-Second Group

	**Overriding_behavior (1 = override)**		
Predictors	*Odds Ratios*	*CI*	*p*
(Intercept)	0.37	0.26 – 0.53	**<0.001**
AI recommendation	0.87	0.65 – 1.16	0.340
**Random Effects**			
σ^2	3.29		
$\tau_{00\ prob\_number}$	0.52		
ICC	0.14		
N_{prob_number}	24		
Observations	960		
Marginal R^2 / Conditional R^2	0.001 / 0.137		

Table S13. *Overriding Behavior in the AI-First Group. Overriding Behavior was calculated as the comparison between the AI prediction and the final classification. AI recommendation reflects the advice from the AI (i.e. "normal"=0, and "abnormal"=1).*

Logistic Model Predicting Overriding Behavior in AI-First Group

	**Overriding_behavior (1 = override)**		
Predictors	*Odds Ratios*	*CI*	*p*
(Intercept)	0.32	0.24 – 0.43	**<0.001**
AI recommendation	2.08	1.56 – 2.76	**<0.001**
**Random Effects**			
σ^2	3.29		
$\tau_{00\ prob\_number}$	0.20		
ICC	0.06		
N_{prob_number}	24		
Observations	960		
Marginal R^2 / Conditional R^2	0.037 / 0.092		

Sample Characteristics

To assess the comparability of the two experimental groups (AI-First and AI-Second), characteristics of participants assigned to the conditions are summarized in Table S14. The groups were similar with respect to age, gender, clinical experience, self-reported auscultation skill, prior experience with digital auscultation, professional position, and their affinity for technology interaction (ATI)[1]. No statistically significant differences were observed between groups for any reported characteristic, indicating successful randomization and excluding alternative explanations for our reported results.

Table S14. *Demographic characteristics, clinical experience, self-reported auscultation skill, prior experience with digital auscultation, professional position, and affinity for technology interaction (ATI) are shown separately for the AI-First and AI-Second groups.*

**Characteristic**	**AI-First**<br>**N = 24**[1]	**AI-Second**<br>**N = 24**[1]	***p*-value**[2]
**Gender**			0.4
male	14 (58%)	17 (71%)	
female	10 (42%)	7 (29%)	
**Age**	29 (26, 35)	29 (26, 36)	0.6
**Experience**	1 (0, 5)	1 (0, 8)	0.8
**Auscultation Skill**			> 0.9
2	5 (21%)	7 (29%)	
3	14 (58%)	12 (50%)	
4	4 (17%)	4 (17%)	
5	1 (4.2%)	1 (4.2%)	
**Previous experience with digital auscultation**			0.2
Yes	6 (25%)	2 (8.3%)	
No	18 (75%)	22 (92%)	
**Position**			0.8
Practical Year	8 (33%)	10 (42%)	
Resident Physician	11 (46%)	8 (33%)	
Specialized Physician	3 (13%)	3 (13%)	
Chief Physician	1 (4.2%)	0 (0%)	

Private Practitioners	1 (4.2%)	2 (8.3%)	
Senior physician	0 (0%)	1 (4.2%)	
ATI Scale	3.94 (3.28, 4.67)	3.94 (3.28, 4.61)	> 0.9

[1] n (%); Median (Q1, Q3)[2] Pearson's Chi-squared test; Wilcoxon rank sum test; Fisher's exact test

Correlation Table

The correlation table is provided on the following page.

Table S15. *Correlation Table. Means, standard deviations, and correlations with confidence intervals*

Variable	*M*	*SD*	1	2	3	4	5	6	7	8	9	10	11	12	13	14	15	16	17	18	19
1. Age	35.48	16.04																			
2. Gender	1.35	0.48	-.17 [-.44, .12]																		
3. Auscultation Skill	3.00	0.77	.20 [-.08, .46]	-.11 [-.39, .18]																	
4. Work experience	8.19	14.33	.99** [.98, .99]	-.12 [-.39, .17]	.19 [-.10, .45]																
5. Dig. Ausc. Exp.	1.83	0.38	.11 [-.18, .38]	-.14 [-.40, .15]	-.37* [-.59, -.09]	.10 [-.19, .37]															
6. Group	1.50	0.51	-.03 [-.31, .26]	-.13 [-.40, .16]	-.05 [-.33, .23]	-.07 [-.34, .22]	.22 [-.06, .48]														
7. Performance _before	0.76	0.08	-.36 [-.67, .05]	-.28 [-.61, .14]	-.11 [-.50, .30]	-.38 [-.68, .03]	.15 [-.27, .52]	NA [NA, NA]													
8. Performance _after	0.70	0.09	-.00 [-.29, .28]	-.02 [-.30, .27]	.23 [-.06, .48]	.03 [-.26, .31]	-.08 [-.36, .21]	-.05 [-.33, .23]	.63** [.30, .82]												
9. Confidence _before	3.14	0.46	.08 [-.33, .47]	-.18 [-.55, .24]	.37 [-.04, .67]	.09 [-.32, .48]	-.34 [-.65, .08]	NA [NA, NA]	.11 [-.31, .49]	.29 [-.13, .62]											
10. Confidence _after	3.22	0.37	-.17 [-.43, .12]	-.19 [-.45, .10]	.22 [-.07, .47]	-.18 [-.44, .11]	-.11 [-.38, .18]	.14 [-.15, .41]	-.03 [-.43, .38]	.13 [-.16, .40]	.83** [.64, .92]										
11. Usefulness	3.32	0.63	-.28 [-.52, .01]	.08 [-.21, .36]	-.35* [-.57, -.07]	-.29* [-.53, -.00]	.30* [.01, .54]	.18 [-.11, .44]	-.13 [-.51, .29]	-.55** [-.72, -.32]	-.36 [-.67, .05]	-.09 [-.36, .20]									
12. Trust	3.36	0.72	-.25 [-.50, .04]	.08 [-.21, .35]	-.37** [-.59, -.10]	-.28 [-.52, .00]	.26 [-.03, .50]	.28 [-.01, .52]	-.10 [-.48, .32]	-.54** [-.71, -.30]	-.27 [-.61, .15]	-.03 [-.31, .26]	.88** [.79, .93]								
13. Replays _before	1.19	0.24	.00 [-.40, .40]	-.28 [-.61, .14]	.29 [-.13, .62]	-.03 [-.43, .37]	.08 [-.33, .47]	NA [NA, NA]	.27 [-.15, .61]	.31 [-.11, .63]	.33 [-.08, .65]	.01 [-.39, .41]	.02 [-.38, .42]	-.06 [-.45, .35]							
14. Replays _after	1.24	0.20	-.11 [-.39, .18]	-.29* [-.53, -.00]	.12 [-.17, .39]	-.11 [-.38, .18]	.18 [-.11, .44]	-.06 [-.33, .23]	-.01 [-.42, .39]	.02 [-.26, .31]	.13 [-.29, .51]	.19 [-.10, .45]	.05 [-.23, .33]	-.07 [-.35, .22]	.43* [.03, .71]						
15. Qual 1	1.88	0.33	.13 [-.16, .40]	.02 [-.27, .30]	.00 [-.28, .28]	.12 [-.17, .39]	-.17 [-.43, .12]	.00 [-.28, .28]	-.36 [-.67, .05]	-.34* [-.57, -.06]	.15 [-.27, .52]	-.01 [-.29, .28]	.06 [-.23, .33]	.04 [-.25, .32]	-.05 [-.45, .36]	-.04 [-.32, .25]					
16. Qual 2	1.62	0.49	.15 [-.14, .42]	.12 [-.17, .39]	-.11 [-.38, .18]	.15 [-.14, .41]	.00 [-.28, .28]	.09 [-.20, .36]	-.39 [-.69, .01]	-.30* [-.54, -.02]	-.03 [-.43, .38]	-.00 [-.29, .28]	-.09 [-.37, .20]	-.12 [-.39, .17]	-.01 [-.41, .40]	-.27 [-.52, .01]	.49** [.24, .68]				
17. Trust Propensity	2.62	0.71	-.15 [-.41, .14]	.17 [-.12, .43]	-.17 [-.43, .12]	-.18 [-.44, .11]	.00 [-.28, .28]	.06 [-.23, .34]	-.29 [-.62, .13]	-.55** [-.72, -.32]	-.37 [-.67, .04]	-.19 [-.45, .10]	.55** [.31, .72]	.63** [.42, .77]	-.07 [-.46, .34]	-.17 [-.43, .12]	.01 [-.28, .29]	.14 [-.15, .41]			
18. Trust Scale	2.75	0.60	-.29* [-.53, -.01]	.31* [.03, .54]	-.22 [-.47, .07]	-.30* [-.53, -.01]	-.03 [-.31, .26]	.03 [-.26, .31]	-.29 [-.62, .13]	-.55** [-.72, -.32]	-.33 [-.65, .08]	-.17 [-.43, .12]	.64** [.43, .78]	.70** [.52, .82]	-.12 [-.50, .30]	-.01 [-.29, .28]	.11 [-.18, .38]	.02 [-.26, .30]	.77** [.63, .87]		
19. Usefulness Scale	4.12	1.58	-.29* [-.53, -.01]	.18 [-.11, .44]	-.28 [-.52, .00]	-.34* [-.57, -.06]	.03 [-.25, .31]	.19 [-.10, .45]	.04 [-.37, .44]	-.40** [-.62, -.13]	-.46* [-.73, -.07]	-.15 [-.41, .14]	.54** [.30, .71]	.65** [.45, .79]	-.25 [-.60, .17]	-.12 [-.39, .17]	-.03 [-.31, .25]	-.14 [-.40, .15]	.56** [.33, .73]	.67** [.48, .80]	
20. ATI	3.92	0.99	-.18 [-.44, .11]	-.42** [-.63, -.15]	.25 [-.04, .50]	-.23 [-.48, .06]	-.11 [-.38, .18]	.05 [-.24, .33]	.29 [-.13, .62]	.03 [-.26, .31]	.30 [-.12, .63]	.18 [-.11, .44]	-.12 [-.39, .17]	-.11 [-.39, .18]	.47* [.08, .73]	.08 [-.21, .36]	.06 [-.23, .34]	-.00 [-.29, .28]	.03 [-.25, .31]	-.06 [-.34, .23]	-.08 [-.36, .21]

Note. M and *SD* are used to represent mean and standard deviation, respectively. Values in square brackets indicate the 95% confidence interval for each correlation. The confidence interval is a plausible range of population correlations that could have caused the sample correlation (Cumming, 2014). * indicates p < .05. ** indicates p < .01. The suffix "_before" refers to baseline measures recorded only in the AI-second group before receiving a system recommendation; "_after" refers to measures taken after the recommendation, available in both groups. "Usefulness" and "Trust" refer to single-item assessments collected during each trial. "Usefulness Scale" (Venkatesh et al.) and "Trust Scale" (Körber) refer to standardized questionnaires administered after the main experiment together with the Affinity for Technology Interaction ("ATI"; Franke et al.). Trust Propensity denotes the respective subscale from Körber's trust questionnaire.

References

1. Franke, T., Attig, C. & Wessel, D. A personal resource for technology interaction: Development and validation of the affinity for technology interaction (ATI) scale. *Int. J. Human–Computer Interact.* 1–12 (2018) doi:10.1080/10447318.2018.1456150.
2. Körber, M. Theoretical considerations and development of a questionnaire to measure trust in automation. *Proc. 20th Congr. Int. Ergon. Assoc. IEA 2018* 13–30 (2019) doi:10.1007/978-3-319-96074-6_2.
3. Venkatesh, V. & Davis, F. D. A Theoretical Extension of the Technology Acceptance Model: Four Longitudinal Field Studies. *Manag. Sci.* **46**, 186–204 (2000).
4. Oliveira, J. *et al.* The CirCor DigiScope dataset: From murmur detection to murmur classification. *IEEE J. Biomed. Health Inform.* 1–1 (2021) doi:10.1109/JBHI.2021.3137048.
5. Vukanovic-Criley, J. M. *et al.* Competency in cardiac examination skills in medical students, trainees, physicians, and faculty: A multicenter study. *Arch. Intern. Med.* **166**, 610–616 (2006).
6. Oliveira, J. *et al.* The CirCor DigiScope Phonocardiogram Dataset. PhysioNet https://doi.org/10.13026/tshs-mw03. (2022).
7. Wickham, H. *et al.* Welcome to the tidyverse. *J. Open Source Softw.* **4**, 1686 (2019).
8. Enzmann, J. A. I. R. source code and/or documentation written by D., Schwartz, M., Jain, N. & Kraft, and S. descr: Descriptive Statistics. (2023).
9. Lüdecke, D. sjmisc: Data and Variable Transformation Functions. *J. Open Source Softw.* **3**, 754 (2018).
10. Lüdecke, D. sjlabelled: Labelled Data Utility Functions (Version 1.2.0). Preprint at https://doi.org/10.5281/zenodo.1249215 (2022).

11. Lüdecke, D. sjstats: Statistical Functions for Regression Models (Version 0.18.2). Preprint at https://doi.org/10.5281/zenodo.1284472 (2024).
12. Lüdecke, D. sjPlot: Data Visualization for Statistics in Social Science (Version 2.8.15). Preprint at https://CRAN.R-project.org/package=sjPlot (2023).
13. Lüdecke, D. *et al.* easystats: Framework for Easy Statistical Modeling, Visualization, and Reporting. *CRAN* https://doi.org/10.32614/CRAN.package.easystats (2022) doi:10.32614/CRAN.package.easystats.
14. Wickham, H. *Ggplot2: Elegant Graphics for Data Analysis*. (Springer-Verlag New York, 2016).
15. Schloerke, B. *et al.* GGally: Extension to ‘ggplot2’ (Version 2.2.0). (2023).
16. Fox, J. & Weisberg, S. *An R Companion to Applied Regression*. (Sage, Thousand Oaks CA).
17. Revelle, W. psych: Procedures for Psychological, Psychometric, and Personality Research (Version 2.3.9). Northwestern University (2023).
18. Kassambara, A. ggpubr: ‘ggplot2’ Based Publication Ready Plots (Version 0.6.0). (2023).
19. Lenth, R. V. emmeans: Estimated Marginal Means, aka Least-Squares Means (Version 1.8.9). (2023).
20. Stanley, D. apaTables: Create American Psychological Association (APA) Style Tables. (2021).
21. Ushey, K., Allaire, J. J., Wickham, H., Ritchie, G. & RStudio. rstudioapi: Safely Access the RStudio API. (2023).
22. Ben-Shachar, M. S., Lüdecke, D. & Makowski, D. effectsize: Estimation of Effect Size Indices and Standardized Parameters. *J. Open Source Softw.* **5**, 2815 (2020).
23. Wickham, H., François, R., Henry, L., Müller, K. & Vaughan, D. dplyr: A Grammar of Data Manipulation. (2023).

24. Pasek, J., Tahk, A., Culter, G. & Schwemmle, M. weights: Weighting and Weighted Statistics. (2021).

25. Bates, D., Mächler, M., Bolker, B. & Walker, S. Fitting Linear Mixed-Effects Models Using lme4. *J. Stat. Softw.* **67**, 1–48 (2015).

26. Kuznetsova, A., Brockhoff, P. B. & Christensen, R. H. B. lmerTest Package: Tests in Linear Mixed Effects Models. *J. Stat. Softw.* **82**, 1–26 (2017).

27. Navarro, D. Learning statistics with R: A tutorial for psychology students and other beginners. (Version 0.6). University of New South Wales (2015).

28. Kassambara, A. rstatix: Pipe-Friendly Framework for Basic Statistical Tests. (2023).

29. Wickham, H. *et al.* readxl: Read Excel Files. (2025).

30. Sjoberg, D. D., Whiting, K., Curry, M., Lavery, J. A. & Larmarange, J. Reproducible Summary Tables with the gtsummary Package. *R J.* **13**, 570–580 (2021).

31. Singmann, H. *et al.* afex: Analysis of Factorial Experiments. (2024).

32. Ryan, K., Yang, H.-J., Kim, B. & Kim, J. P. Assessing the impact of AI on physician decision-making for mental health treatment in primary care. *Npj Ment. Health Res.* **4**, 16 (2025).
